# Targeting FAM83D triggers tumor cell senescence via cGAS-STING signaling activation and reprograms TAMs to combat glioma

**DOI:** 10.1186/s13046-026-03681-y

**Published:** 2026-02-26

**Authors:** Hongwei Liu, Xuelei Lin, Luohuan Dai, Wei Zhang, Yihao Zhang, Na Liu, Yueshuo Li, Jens Jeshu Peters, Jia Gu, Kang Peng, Nian Jiang, Siyi Wanggou, Xuejun Li

**Affiliations:** 1 Department of Neurosurgery, Xiangya Hospital, Central South University, Changsha, Hunan 410008 China; 2 Hunan International Scientific and Technological Cooperation Base of Brain Tumor Research, Xiangya Hospital, Central South University, Changsha, Hunan 410008 China; 3 National Clinical Research Center for Geriatric Diseases (Xiangya Hospital), Changsha, China; 4 Department of Neurosurgery, The Second Affiliated Hospital of Hainan Medical University, Haikou, Hainan 570311 China; 5 Department of Anatomy and Neurobiology, School of Basic Medical Science, Central South University, Changsha, Hunan 410013 China; 6 Cancer Research Institute, Xiangya School of Medicine, Central South University, Changsha, Hunan 410078 China; 7 Department of Radiology, Xiangya Hospital, Central South University, Changsha, Hunan 410008 China

**Keywords:** Glioma, Cellular senescence, FAM83D, cGAS-STING signaling, Macrophage polarization

## Abstract

**Background:**

Glioma, a prevalent and aggressive primary brain tumor, has a poor prognosis despite the administration of standard treatment. Cellular senescence is thought to have a good protective effect in limiting the malignant progression of tumors, while the senescence-associated secretory phenotype produced by senescent cells may influence the activity of immune cells in the microenvironment. Macrophages, as a major component of the glioma microenvironment, play an important role in regulating the innate and adaptive immune responses which is essential for tumor suppression. Therefore, identifying potential targets that connect tumor cell senescence with macrophage reprogramming may facilitate the development of new therapeutic agents in glioma.

**Methods:**

Here, we developed the CellToAge algorithm and leveraged high-throughput sequencing data to map senescence-associated signatures in glioma. Based on in vitro and in vivo experiments including the cell co-culture model, in situ allograft mouse model, and single-cell transcriptome sequencing, we further elucidated the interplay between tumor cell senescence and macrophage polarization induced by knockdown of *FAM83D* in glioma.

**Results:**

Notably, we identified the projected senescence-associated signatures in glioma (PSAG) and focused on the potential target FAM83D. In vitro and in vivo assays showed that knockdown of *FAM83D* resulted in abnormal cell division, which increased double-stranded DNA in the cytoplasm, thereby inducing tumor cell senescence by activating the cGAS-STING signaling to suppress glioma progression. Synchronously, knockdown of *FAM83D* could also induce glioma cells to produce a specific senescence-associated secretory phenotype (SASP), which promoted the senescence of neighboring tumor cells and drove macrophage polarization toward an M1 state. Additionally, ANXA1-FPR1/2 ligand-receptor signaling involved in tumor cells-macrophages crosstalk was associated with macrophage polarizations, which was further validated based on our clinical glioma cohort.

**Conclusions:**

Our data revealed the critical role of FAM83D in regulating tumor cell senescence and shaping the tumor microenvironment with a specific impact on macrophage polarization. These insights open avenues for targeted therapeutic strategies aimed at modulating the FAM83D-cGAS/STING-SASP-TAMs axis in the management of glioma.

**Supplementary Information:**

The online version contains supplementary material available at 10.1186/s13046-026-03681-y.

## Background

Glioma is one of the most common primary tumors in the central nervous system, accounting for 30% of all primary brain tumors and 80% of malignant brain tumors [1]. It is the leading cause of death from primary brain tumors, with a median survival of 15–18 months for patients [1, 2]. Due to a deeper understanding of the underlying molecular biology of glioma and its interaction with the immune system, immunotherapies such as immune checkpoint inhibitors, CAR-T cell therapy, and tumor vaccines have become new approaches to treat glioma in addition to standard treatment [3–5]. Despite the continuous progress in the treatment of glioma, these treatments have not yet shown good clinical efficacy in glioma patients. The limited benefits of these therapies can be attributed to genetic, epigenetic, and microenvironmental factors that affect cell biological activities and drive tumor heterogeneity [6]. Identifying potential targets related to the above factors may help to further understand the mechanism of glioma progression and develop new therapeutic drugs.

With the deepening of cancer research in recent years, researchers have refined the enormous complexity of cancer phenotypes and genotypes into several related cancer characteristics, such as maintaining proliferation signals, escaping growth inhibition, and resisting cell death [7]. As a complementary mechanism to programmed cell death, cellular senescence, a typical form of irreversible proliferation arrest, can remove pathological, dysfunctional, or other unnecessary cells at the appropriate time [8]. It has a series of overlapping characteristics with cancer, such as genomic instability, epigenetic changes, and chronic inflammation [8]. For a long time, cell senescence has been considered to have a good protective effect in limiting the malignant progression of tumors; that is, tumor cells are induced to undergo senescence [9]. However, there is also evidence that the senescence-associated secretory phenotype (SASP) produced by senescent cells involves the release of a large number of bioactive proteins, including chemokines, cytokines, and proteases, which can transmit signaling molecules in a paracrine manner to adjacent tumor cells and other cells in the tumor microenvironment, playing different roles in inhibiting and promoting tumor growth [10]. Whether the heterogeneity of these senescent cells triggering pro-tumor or anti-tumor activities is regulated by different senescent cell subpopulations or determined by the different tumor microenvironments remains to be further explored. In glioma, metformin and simvastatin have been reported to have anti-tumor effects by inducing the senescence state of tumor cells and regulating the expression of key genes involved in the SASP [11]. Although many promising anti-senescence drugs have been discovered, and some of them have even entered clinical trials, prospective results of large-scale tumor cohort trials are still to be reported [12]. Therefore, identifying reliable markers related to tumor cell senescence in glioma and developing drugs based on these targets has significant clinical translational value.

The family with sequence similarity (FAM) gene family includes a group of genes that have not been fully characterized but have similar protein sequences. Some studies have reported that FAM family genes play a key role in the pathophysiology of various diseases, including proliferation, invasion, migration, and drug resistance [13–16]. In addition, specific members of the FAM gene family, such as FAM38A, which encodes the mechanosensitive ion channel protein PIEZO1 and can sense and transmit the mechanical stiffness of tissues to promote the progression of glioma, are considered to be new targets for the treatment of solid tumors [17]. Among them, FAM83D (Family with sequence similarity 83 member D), also known as the spindle protein CHICA, is initially shown to participate in the positive regulation of the G1/S phase transition, protein localization of the mitotic spindle, and intracellular signal transduction by interacting with microtubule-associated proteins [18, 19]. Abnormal mitosis leading to cell cycle arrest and cell morphological changes is one of the hallmarks of senescent cells, suggesting a possible link between FAM83D and cellular senescence. Currently, studies have shown that downregulating FAM83D can inhibit glioma proliferation and invasion by regulating the AKT/mTOR and AKT/Wnt signaling pathways [20, 21]. However, the specific relationship between FAM83D and cellular senescence and its mechanism involved in glioma progression requires further investigation.

Innate immunity, the first line of defense for mammals against foreign invaders, can clear infections by identifying unique infectious agents and triggering adaptive immunity [22]. Among all known innate immune signaling pathways, the cGAS-STING signaling pathway has attracted widespread attention due to its important role in sensing cytoplasmic DNA, regulating cell senescence, and influencing tumorigenesis [23]. In this pathway, cyclic GMP-AMP synthase (cGAS) is a cytoplasmic DNA sensor that can be activated by binding to double-stranded DNA (dsDNA) [24, 25]. Activated cGAS produces the second messenger cyclic GMP-AMP (cGAMP), which then binds to and activates the stimulator of interferon genes (STING), ultimately leading to the production of type I interferons and inflammatory cytokines [24, 25]. Recent studies have reported that abnormal chromatin fragments released from the nucleus of senescent cells into the cytoplasm can be directly recognized by cGAS, which then activates the production of SASP factors through STING [26, 27]. Considering the importance of FAM83D for chromosome segregation during mitosis and the relationship between the DNA damage response in senescent cells and the cGAS-STING signaling pathway, there may exist an unexpected connection between them.

Macrophages are key mediators of tissue homeostasis. Tumors can reprogram macrophages to stimulate their own proliferation, angiogenesis, and metastasis, which has aroused interest in targeting macrophages for cancer therapy, and their effectiveness has been confirmed in preclinical studies [28]. Tumor-associated macrophages (TAMs) are the main components of the tumor microenvironment (TME) in glioma, accounting for 30% to 50% of the total tumor tissue composition [29]. It includes macrophages derived from blood monocytes and microglia resident in the brain [29]. Currently, the most discussed function of TAMs is the M1-M2 polarization system, in which M1 macrophages exhibit anti-tumor properties while M2 macrophages exhibit pro-tumor properties [30]. Growing evidence shows that TAMs are usually in the M2 state in tumors, and their content is associated with higher tumor grade [31, 32]. In contrast, the polarization of TAMs to the M1 state can suppress tumors by producing pro-inflammatory cytokines and key molecules that stimulate anti-tumor responses of T cells [33]. Considering the complex gene regulation of the SASP, these secretory factors can both produce an immunosuppressive environment or promote immune surveillance and tumor clearance, which is closely related to the function of macrophages [34]. In glioblastoma, autophagy inhibition has been reported to enhance tumor cell senescence and alter the expression profile of the SASP, inducing senescence of adjacent tumor cells and transforming macrophages to an anti-tumor M1 state [35]. This suggests that inducing tumor cell senescence may achieve tumor suppression by affecting the tumor immune and the conversion of M2 TAMs to M1 TAMs through the intrinsic mechanism in glioma TME is still an area to be actively studied.

The similarities between cancer and development have always been of interest. In the 19th century, pathologists noticed morphological similarities between developing tissues and tumor tissues, leading to the hypothesis of “embryonic dormancy” [36]. This suggests that tumor tissues contain embryonic remnants that are usually dormant and may be reactivated to become malignant tumors, even in tissues from older people. One of the most prominent examples is cerebellar tumors in children. By comparing the transcriptional similarities between the developing cerebellum and medulloblastoma based on single-cell RNA sequencing (scRNA-seq), researchers identify a population of tumor cells that is closest to a specific cerebellar developmental time and lineage cell population, which is considered to be the origin of the tumor [37]. Recently, a population of glioma cells, which can mimic the development trajectory of normal astrocyte and oligodendrocyte lineage, has also been found [38, 39]. Therefore, we hypothesize that by mapping the transcriptional profile of glioma to the developing brain, we can indirectly analyze the age heterogeneity of glioma cells and obtain a senescence-related signature at the single-cell level.

Here, we focused on tumor cell senescence and macrophage polarization, combining high-throughput sequencing data with in vitro and in vivo experiments to elucidate the relationship and mechanisms between these two processes in glioma. We reported a projected senescence associated signatures in glioma (PSAG), which was closely associated with the regulation of glioma cell senescence. We further validated the role of potential target FAM83D in the progression of glioma and found that knockdown of *FAM83D* could induce glioma cell senescence by activating the cGAS-STING signaling. Additionally, knockdown of *FAM83D* in glioma cells reshaped the TME and promoted M1 polarization of TAMs via tumor cells-TAMs crosstalk. These findings were also confirmed by an independent glioma cohort analysis. Our data suggest that FAM83D has the potential to become a mature biomarker and therapeutic target in glioma.

## Methods

### Glioma cohort recruitments

One cohort of 132 patients with glioma receiving surgery at Xiangya Hospital, Central South University, was recruited for RNA-seq. Totally, 132 glioma tissue samples and 38 adjacent normal tissue samples were included. These samples were immediately stored in liquid nitrogen after surgical resection. Clinical data for all patients were collected from medical records retrospectively, including gender, age, pathology, and tumor grade. All patients gave written informed consent, and the Medical Ethics Committee at Xiangya Hospital, Central South University, approved this study.

### Cell culture

Human normal astrocytes (HA1800), glioma-derived cell lines (A172, U87, U251, LN229, and GL261), and a mouse microglial cell line (BV2) were obtained from the Cancer Research Institute of Central South University. HA1800 were cultured using Astrocyte Medium (ScienCell) supplemented with 1% Astrocyte Growth Supplement and 10% fetal bovine serum (FBS). Glioma-derived cell lines and a microglial cell line were cultured in DMEM (Bio-Channel) supplemented with 10% FBS. All cells were maintained at 37 °C with 5% CO_2_.

### Lentivirus transfection and generating stable cell lines

*FAM83D* knockdown lentivirus (shFAM83D #1: GATCTGAAAGTTCATCCTGAA, shFAM83D #2: CCTGACTTTGTCACCTTTGTT) was designed and synthesized by GENECHEM (Shanghai, China). The targeted sequences of *FAM83D* were cloned into the GV112 vector, and an empty vector (shControl) was used as a negative control. Cells were transfected with lentivirus for 24 h and selected for positive cells with 2 µg/ml puromycin.

### Real-time quantitative PCR

Total RNAs for tissue samples or cell lines were extracted according to the manufacturer’s instructions of the Total RNA rapid extraction kit (GeneBetter). And then, 1 µg of total RNA was reverse transcribed into cDNA using TransScript^®^ Uni All-in-One cDNA Synthesis Kit (TransGen). Real-time quantitative PCR (RT-qPCR) was performed on LightCycler^®^ 96 Instrument (Roche) with 2× BRYT Green qPCR Master Mix (Promega). Designed primers were listed in Table S1. GAPDH was used as an internal control for gene expression, and the ΔΔCT method was utilized to quantify relative mRNA expression.

### Immunoblotting

Total protein for tissue samples or cell lines was extracted using RIPA buffer supplemented with protease and phosphatase inhibitors (Beyotime). The protein concentration was measured by the BCA method, and approximately 15–30 µg of total proteins were loaded and analysed by Western blots (WB). The primary antibodies used for WB were mouse anti-GAPDH (Abclonal, no. AC033, 1:10,000) and rabbit anti-FAM83D (Abcam, no. ab236882, 1:500), rabbit anti-p21^WAF1/CIP1^ (Cell Signaling Technology, no. 2947 S, 1:1000), rabbit anti-p16^INK4a^ (Selleckchem, no. F3773, 1:1000), rabbit anti-p65 (Selleckchem, no. F0006, 1:1000), and rabbit anti-phospho-p65 (Selleckchem, no. F0155, 1:1000). Secondary antibodies used for WB were HRP-conjugated goat anti-rabbit IgG (Servicebio, no. GB23303, 1:5000) and HRP-conjugated goat anti-mouse IgG (Servicebio, no. GB23301, 1:10,000). Finally, immunoreactive bands were visualized using the Bio-Rad Chemidoc imaging system and quantified using ImageJ software.

### Immunofluorescence staining

After seeding cells in coverslips with an appropriate number, cells were fixed with 4% paraformaldehyde (PFA) for 15 min and permeabilized using 0.1% TritonX-100 for 15 min at room temperature. Then, the blocking buffer (5% bovine serum albumin) was applied for 1 h at room temperature. Cells were incubated with rabbit anti-FAM83D antibody (Abcam, no. ab236882, 1:100), mouse anti-Ki67 antibody (Cell Signaling Technology, no. 9449 S, 1:1000), rabbit anti-γH2AX antibody (Cell Signaling Technology, no. 9718 S, 1:500), mouse anti-α-Tubulin antibody (Cell Signaling Technology, no. 3873 S, 1:1000), mouse anti-dsDNA antibody (Santa Cruz Biotechnology, no. sc-58749, 1:50), rabbit anti-STING antibody (Proteintech, no. 19851-1-AP, 1:100), rabbit anti-Cd206 antibody (Proteintech, no. 18704-1-AP, 1:500) or rabbit anti-Cd86 antibody (Abclonal, no. A1199, 1:100) overnight at 4 °C. All antibodies were diluted in 1% blocking buffer. Secondary antibody conjugated to Alexa Fluor 555 or 488 (Invitrogen A32794, A32766) was used at 1:1000 for 1 h in the dark at room temperature. Finally, DAPI was added to stain the nucleus and incubated in the dark for 5 min. Images were taken using a Leica DM4B or a STELLARIS 5 microscope and quantified using ImageJ software.

### SA-β-gal staining

Senescence-associated β-galactosidase (SA-β-gal) staining was performed in accordance with the manufacturer’s instructions (Beyotime, no. C0602). Firstly, an appropriate number of cells were inoculated into a 6-well plate and incubated at 37 °C until the cells in the plate reached an appropriate density. The medium was pure culture media supplemented with additional STING inhibitor H-151 (Selleckchem, no. S6652, 1 µM) or Dimethyl sulfoxide (DMSO). Then, the cells were fixed with 4% PFA and stained with β-galactosidase staining solution at 37 °C overnight. Finally, images were taken using a Leica DM4B microscope and quantified using ImageJ software.

### Colony formation assay

One thousand cells were seeded into a 6-well plate and maintained at 37 °C for 7–14 days by routine culture. The colonies were fixed with 4% PFA and stained with 0.1% crystal violet. Images were taken using a Leica DM4B microscope and quantified using ImageJ software.

### Flow cytometry

Cell cycle arrest was analyzed by flow cytometry in accordance with the manufacturer’s instructions (Beyotime, no. C1052). Briefly, an appropriate number of cells were trypsinized and fixed with ice-cold 70% ethanol at 4 °C overnight. After fixation, cells were incubated with propidium iodide (PI) staining solution for 30 min in the dark at room temperature. Then we used the DxP Athena flow cytometry system to detect fluorescence. Quantification of the percentage of cells in G0/G1, S, and G2/M phases was performed using the FlowJo software (v10.8.1).

### Conditioned media collection

Conditioned media from U251 and LN229 cells treated with the lentiviruses were used to investigate the effects of the SASP on wild-type cells. U251 and LN229 cells from the various treatment groups were seeded at a density of 1 × 10^5^ cells per well in 6-well plates. After 48 h of routine culture, the cell culture medium was collected and centrifuged at 1000 g for 5 minutes to precipitate cell debris and impurities. The supernatant was aspirated as the conditioned media. This conditioned media was used to culture wild-type tumor cells or microglia and can be stored at -20 °C.

### Co-culture of tumor cells and microglia

An appropriate number of BV2 cells were prepared and inoculated into the 12-well plate. Then a cell culture chamber with a pore size of 0.4 μm (NEST, no. 724122) was placed into the 12-well plate and the same number of U251 and LN229 cells with different treatments were seeded into the chamber. After culturing at 37 °C for 48 h, BV2 cells were obtained for further experiments.

### In vivo experiments

In total, 4 × 10^4^ GL261 cells were orthotopically injected into 6–8-week-old female C57BL mice. The coordinates were 1.5 mm lateral to midline, 2 mm posterior to bregma, and − 2.5 mm deep to the cranial surface. Mice were given a 7-day recovery period and kept in a sterile environment under a 12/12-h light/dark cycle, 21–23 °C, and 40–60% humidity unless specified otherwise. Starting from the 7th day, in vivo bioluminescence imaging was used to assess tumor growth by the Fusion system (Vilber Lourmat, France). The end of the mice’s life was determined based on the tumor size and the overall health condition of the mice. The mice’s brain tissue between different groups on day 14 was dissected for scRNA-seq and prepared for paraffin sections at a thickness of 5 μm. These sections were then used for Hematoxylin-eosin (H&E) and multiplex immunohistochemistry staining.

### Multiplex immunohistochemistry

Paraffin-embedded tissue sections were deparaffinized and rehydrated using an environmentally friendly dewaxing solution and ethanol. Then heat-induced epitope retrieval was performed, followed by blocking with 3% hydrogen peroxide solution for 25 min and 10% rabbit serum or 3% BSA for 30 min. Finally, we used a multiplex immunohistochemistry (mIHC) kit (TSA PLus multi-fluorescent dyeing kit, Servicebio) to stain Iba1 (Servicebio, no. GB12105-100, 1:500), iNOS (Servicebio, no. GB11119-100, 1:500), and Cd206 (Servicebio, no. GB113497-100, 1:300) in tissue sections. Briefly, after incubating the first primary antibody at 4 °C overnight, the slide was incubated with the corresponding HRP labeled secondary antibody at room temperature for 50 min, and then incubated with the corresponding type of TSA working solution at room temperature for another 10 min. Subsequently, the sections were subjected to antigen retrieval again to prepare the next round of staining. Lastly, DAPI was used to mark the nucleus, and we used a Nikon Eclipse C1 microscope and a Pannoramic MIDI scanner system for multispectral slice analysis under the appropriate fluorescent filters.

### Public datasets retrieval and processing

Two human glioma single-cell datasets (GSE117891 and GSE182109) [40, 41], one human brain development single-cell dataset (GSE120046) [42], and one mouse brain aging single-cell dataset (GSE129788) [43] were obtained from the Gene Expression Omnibus (GEO) database. We first used the R package Seurat (v4.0.3) [44] to perform data normalization and remove low-quality cells. Then principal component analysis (PCA) was performed based on the top 3000 features with the highest variability, and Uniform Manifold Approximation and Projection (UMAP) was used for dimensionality reduction and visualization. The annotation of cell types in each dataset was derived from the original research. Ultimately, we generated two human glioma atlases, each containing 5,165 cells (GSE117891) and 225,168 cells (GSE182109), a human brain development atlas containing 12,343 cells (GSE120046), and a mouse brain aging atlas containing 37,069 cells (GSE129788).

Normalized spatial transcriptome data of glioma and their corresponding phenotypic information were obtained from a previously published study [45]. We used the R package Seurat to visualize and analyze the data. The distribution of benign and malignant meta-programs (MPs) and tissue layers corresponding to each spot on the spatial transcriptome was derived from the original research. Due to the sparsity of single-cell and spatial transcriptome sequencing data, we defined cells with a raw gene count > 0 as gene-positive cells for statistical analysis.

Processed TCGA glioma RNA-seq, including lower grade glioma (LGG) and glioblastoma (GBM), and GTEx RNA-seq with their clinical data, were downloaded from the University of California, Santa Cruz (UCSC) Xena through the online database website (https://xenabrowser.net/). Two batches of the glioma RNA-seq in the Chinese Glioma Genome Atlas (CGGA), including CGGA_325 and CGGA_693 with their clinical data, were obtained from the CGGA website (http://www.cgga.org.cn/). Another glioma cohort (GSE16011) was obtained from the GEO database. We excluded samples without complete survival information from these four glioma cohorts. All transcriptome data used for visualization were normalized by Log2 (TPM + 1) transformation.

### Preparation of bulk RNA sequencing library and process

RNA extracted from glioma tissue samples or cell lines was used to construct transcriptome libraries using Novogene’s Illumina sequencing platform or BGI’s DNBseq platform. After sequencing, the raw data were filtered and quality controlled to obtain clean data. We used HISAT2 software (v2.2.1) to align data to the human reference genome (GRCh38), and then applied StringTie software (v2.1.4) to assemble transcripts and quantify gene expression levels. All transcriptome data used for visualization were normalized by Log2 (TPM + 1) transformation.

### Preparation of a single-cell sequencing library and process

Freshly resected tumor specimens from different mice with the same treatment (3 mice in each group) were mixed together and dissociated into single-cell suspensions to avoid too few cells. RNA was extracted from these single cells, and a single-cell transcriptome library was constructed using the DNBelab C series platform of BGI. Raw sequencing data of scRNA-seq were aligned to the GRCh38 reference genome through DNBelab software (v2.1.3), and then feature-barcode counts matrices were processed using the R package Seurat. Cells with fewer than 500 genes, more than 7500 genes, and more than 10% mitochondrial genes detected were excluded because of low quality. Besides, the R package DoubletFinder (v2.0.3) [46] was used to remove potential doublets. Totally, 41,578 cells were retained for further analysis. After quality control, the scRNA-seq dataset was log normalized and dimensionality reduced by performing PCA. To eliminate potential batch effects, computed principal components (PCs) were batch corrected using the ‘RunHarmony’ function from the R package harmony (v0.1.0) [47] and the top 15 batch-corrected PCs were selected for UMAP embedding generation. The cell clusters were then identified by performing K-nearest neighbors (KNN) unsupervised clustering with a resolution set as 0.3. We used two criteria to categorize every cluster into a known biological cell type: (1) the expression of conventional markers described in previous research and (2) copy number variations (CNVs) of each cell estimated by the inferCNV algorithm (v1.8.0) [48] to distinguish between benign and malignant cells. In subsequent analyses, we extracted each cell type separately and re-clustered it to find subclusters for exploring their heterogeneity.

### Mapping the human glioma atlas to the human brain development atlas

We used the human brain development atlas as a reference and sequentially applied the KNN algorithm, the mutual nearest neighbors (MNN) algorithm, and the shared nearest neighbors (SNN) algorithm using the ‘FindTransferAnchor’ function from the R package Seurat to search for the most relevant cell in the reference atlas for each cell in the human glioma atlas. After establishing anchor points, we used the ‘MapQuery’ function to annotate the cell type and age label from the reference atlas to each cell in the human glioma atlas. Since the embryonic development period from gestational week 7 (GW7) to gestational week 14 (GW14) was considered as an early embryonic stage, while the period from gestational week 16 (GW16) to gestational week 28 (GW28) was considered as a mid-embryonic stage [42], we defined tumor cells with age annotated to GW7 to GW14 as relatively young cells, and the others as relatively old cells.

### Identification of differentially expressed genes

In single-cell transcriptome datasets, we used the ‘FindAllMarkers’ function from the R package Seurat to screen differentially expressed genes (DEGs) between each cell group. For dataset GSE117891, the threshold for identifying DEGs between young and old tumor cells was set as a log2 fold change (Log2FC) > 1.5 and an adjusted *p* value < 0.01, while the threshold for identifying DEGs between normal and tumor cells was set as a Log2FC > 0.5 and an adjusted *p* value < 0.01. For dataset GSE129788, the threshold for identifying DEGs between young and old brain cells in the normal mouse brain was set as a Log2FC > 0.1 and an adjusted *p* value < 0.05. In the scRNA-seq of tumor tissue from tumor-bearing mice, genes with an adjusted *p* value < 0.05 and a Log2FC > 0.5 were defined as statistically significant marker genes for each cell type.

In publicly available bulk glioma datasets, we used the R package limma (v3.48.0) [49] to screen DEGs between two groups, with the threshold set as Log2FC > 1.5 and an adjusted *p* value < 0.01. In the RNA-seq of cell lines, we used the R package DESeq2 (v1.32.0) to identify DEGs between the control and knockdown group based on the raw count matrix. We defined DEGs with an absolute value of Log2FC > 0.5 and an adjusted *p* value < 0.05 as genes with statistical differences.

### Core features selection by machine learning algorithms

The machine learning framework Mime [50, 51] were used to screen core prognostic variables based on the provided transcriptome data and corresponding survival information. The implementation of this module included three steps. Firstly, we preliminarily filtered the features through univariate Cox regression analysis. Then, we used 8 machine learning algorithms, including Lasso, Enet, Boruta, CoxBoost, RSF, eXtreme gradient boosting (Xgboost), StepCox, and support vector machine recursive feature elimination (SVM-REF), to further screen features that were closely related to patients’ prognosis. In this process, we executed the Lasso algorithm 1000 times with different seeds and finally retained those features that were selected more than 50 times. Since the StepCox algorithm included forward, backward, and bidirectional parameters, and Enet involved 9 α parameters (ranging from 0.1 to 0.9), we actually used a total of 18 machine learning models for feature screening. Finally, we defined the features most frequently selected by the machine learning models as core features.

### Identification of MPs based on non-negative matrix factorization

Non-negative matrix factorization (NMF) was a method for analyzing high-dimensional data that allowed for the extraction of sparse and meaningful features from a set of non-negative vectors. Therefore, it was well-suited for decomposing scRNA-seq data, effectively reducing large, complex matrices into a few interpretable genetic programs, called meta-programs (MPs). To resolve intra-tumor heterogeneity and identify consistent gene signatures corresponding to cell states, we used the R package GeneNMF (v0.6.2) to identify their MPs. Considering the large number of MPs, only those containing more than 10 genes were retained. Genes within the MPs with an explanatory power > 0.5 and a confidence score > 0.7 for the NMF weight ratio were selected as features of the MPs. Ultimately, four MPs containing 131 genes were identified. The cellular functional states corresponding to each MP were assessed by the R package clusterProfiler (v4.7.1) [52].

### Cell-cell communication

CellPhoneDB (v2.0.0) [53] was a tool that integrated existing ligand-receptor interactions to analyze cell-cell communication networks at the molecular level. As described above, 41,578 individual cells were first divided into 10 cell types. Then, the software was used to determine the interaction networks between different cell types in different groups respectively. Finally, we selected interaction pairs with a *p* value < 0.05 returned by CellPhoneDB to evaluate the effect of *FAM83D* knockdown on cell-cell communication.

### Survival analysis

According to the median expression level of *FAM83D*, patients were divided into the *FAM83D* high expression group and the low expression group. To determine the prognostic value of *FAM83D*, we used the R package survival (v3.3-1) and survminer (v0.4.9) to calculate hazard ratios (HRs) with 95% confidence intervals (CI) and log-rank *p* values in survival analysis. In addition, the R package ezcox (v1.0.2) was used to perform multivariate Cox proportional hazard regression analysis.

### Gene sets enrichment analysis

Aging-related projects and gene sets were derived from the Aging atlas (https://ngdc.cncb.ac.cn/aging/index) [54]. The transcriptome data of these aging-related projects originated from adipose-derived stem cells induced by *YBX1* knockdown, mesenchymal stem cells induced by *CLOCK* knockdown, *ZKSCAN3* knockdown, or *ALKBH1* knockdown, vascular endothelial cells induced by *FOXO3A* knockdown, and *WRN*-deficient mesenchymal stem cells inhibited by vitamin C treatment. Log2FC values ​​for *FAM83D* were extracted from these six datasets to explore gene expression changes associated with aging. The well-defined pathways, including cellular senescence, stress-induced senescence, cytosolic DNA sensing pathway, and hallmark of cancer, were downloaded from the Molecular Signatures Database (MSigDB, https://www.gsea-msigdb.org/gsea/msigdb/index.jsp) [55]. Then cell cycle related genes in cellular senescence were extracted alone as gene set ‘replicative senescence’. The signatures associated with normal young or old adult brain were identified by performing DEGs analysis on GTEx RNA-seq data of brain, with adults categorized into a young group (aged 20 to 59) and an old group (aged 60 to 79). While the other well-defined gene sets, including stem cell signatures, T-cell functional signatures, macrophage-associated signatures and TME-associated signatures, were accessed from previous studies [56–60].

In scRNA-seq, we calculated gene set enrichment scores for each cell using the ‘AddModuleScore’ function from the R package Seurat. In glioma tissue RNA-seq, we applied the ssGSEA algorithm [61] from the R package GSVA (v1.40.1) to calculate gene set enrichment scores. All scores were scaled to range 0–1. The ligand-receptor signaling of ANXA1-FPP1 and ANXA1-FPP2 (ANXA1/FPR expression) in glioma tissues was determined by the average expression level of these two genes. The M1/M2 signature score was determined by the enrichment ratio of M1 macrophage-associated signatures to M2 macrophage-associated signatures. The pro-tumor/anti-tumor cytokines score was determined by the enrichment ratio of tumor-promoting cytokines to anti-tumor cytokines. Additionally, the enrichment results of GO or KEGG among DEGs were generated by the ‘compareCluster’ function implemented in the R package clusterProfiler.

### Quantification and statistical analysis

All statistical analyses were performed in R (v4.1.3) or Python (v3.6.13). Graphs were generated in Adobe Photoshop software and BioRender.com. Continuous variables between groups were compared using the Wilcoxon rank-sum test, t-test, Kruskal-Wallis test, or ANOVA test based on the distribution of data. Correlations between variables were calculated using Pearson’s correlation or Spearman’s correlation test based on the distribution of data. Categorical variables between groups were compared using Fisher’s exact test or the Chi-Squared test. Immunoblotting and immunohistochemistry data were quantitatively analyzed by ImageJ software. Luciferase signal data were quantitatively analyzed by EvolutionCapt software. Data were further normalized by the mean of the data in the control group. A two-sided *p* value < 0.05 was considered statistically significant. All the statistical tests used in this study were described in the relevant sections and figure legends.

## Results

### Integrated single-cell transcriptomic analysis reveals projected senescence associated signatures in glioma

**Fig. 1:**
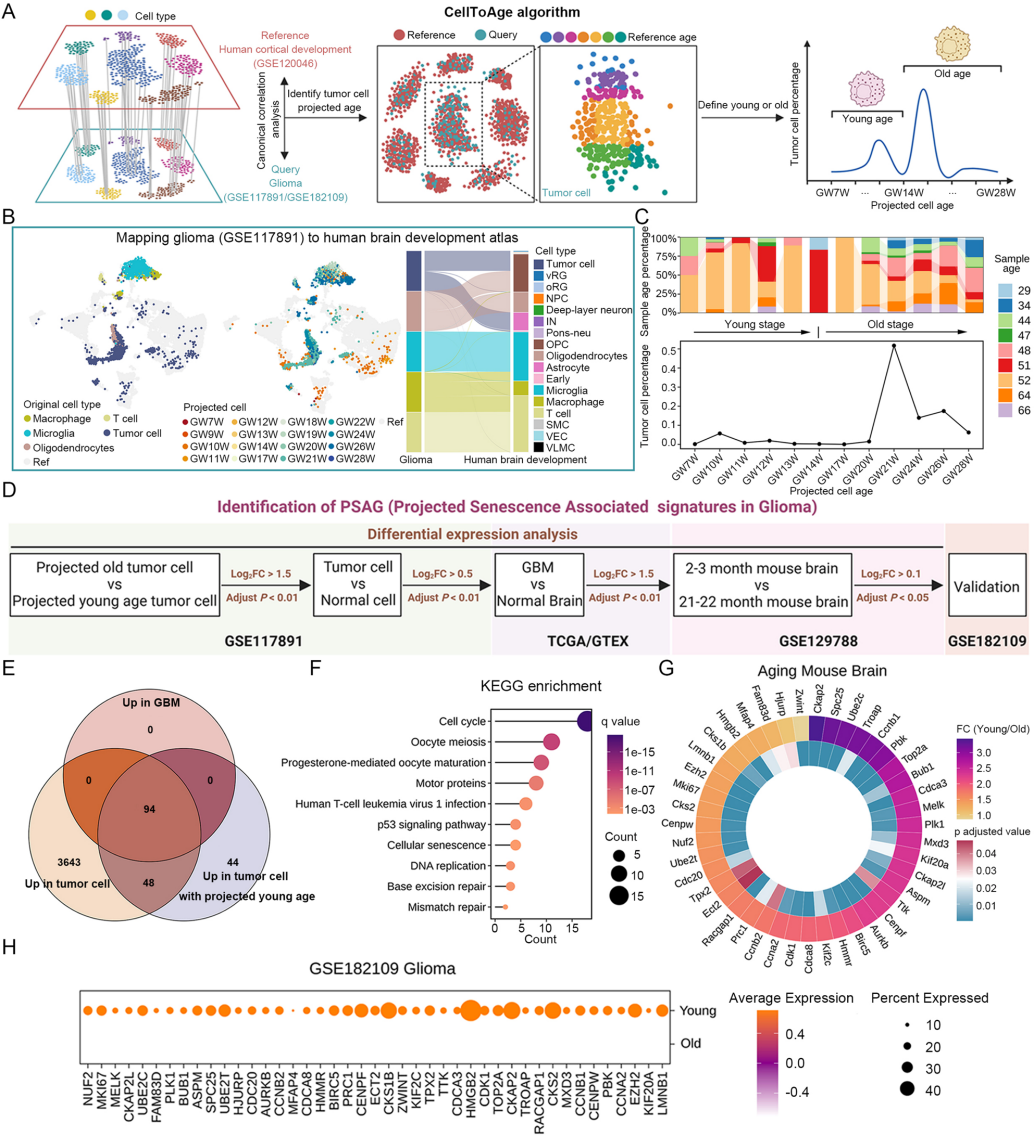
Identification of senescence associated signatures in glioma. **A** Workflow for identifying the relative age of tumor cells based on the CellToAge algorithm. **B** Projected cell types and ages in glioma by mapping the glioma dataset GSE117891 to the human brain developmental atlas. The diagram on the right shows the consistency between the original cell types and projected cell types in the microenvironment. **C** Distribution of projected cell ages in glioma dataset GSE117891. The bar chart at the top indicates the age of the corresponding patients. **D** Workflow showing how to identify projected senescence associated signatures in glioma. **E** Intersection of genes upregulated in relatively young tumor cells within the GSE117891 dataset, tumor cells within the GSE117891 dataset, and GBM tissues within the TCGA cohort. **F** KEGG enrichment based on 94 common genes. *q* value, a corrected *p* value determined by the false discovery rate. **G** Expression patterns of PSAG between young mouse brain cells and aging mouse brain cells in the GSE129788 dataset. *p* adjusted value, a corrected *p* value determined by bonferroni. **H** Expression level of PSAG in the glioma dataset GSE182109 between relatively young and old tumor cells

To uncover the age heterogeneity of glioma cells, we developed a CellToAge algorithm to identify the relative age of tumor cells and classify them into young and old states (Fig. 1A). This algorithm mapped the human glioma atlas to the human brain development atlas (Fig. S1A, B) by comparing intercellular similarities and annotated the developmental age of normal cells onto glioma cells. We found that the mapped cell types within the glioma microenvironment were highly consistent with their own cell types in both glioma datasets (Fig. 1B and S1C). For example, glioma cells primarily resembled astrocytes and oligodendrocytes in the human brain developmental atlas, consistent with their lineage of origin, while immune cells were mapped to immune cells in the human brain developmental atlas, demonstrating the high reliability of our developed algorithm (Fig. 1B and S1C). Interestingly, the mapped developmental age of tumor cells derived from the same tissue or different tissues were varied, reflecting the age heterogeneity of tumor cells (Fig. 1C). Furthermore, some glioma cells from older patients were more similar to cells from earlier developmental stages than those from younger patients (Fig. 1C). In both glioma datasets, a small number of tumor cells were mapped to early developmental stages, which were defined as projected young cells, while the rest were defined as projected old cells (Fig. 1C and S1D). To elucidate the relationship between the projected age of tumor cells and cellular senescence or a developmental-like state, we further compared the enrichment of relevant signatures in projected young versus projected old tumor cells. The results showed that the projected young tumor cells were significantly enriched for signatures related to the regulation of both replicative senescence and stress-induced senescence, but not for stem cell signatures, in both glioma datasets (Fig. S1E). Additionally, the normal young adult brain associated signature was significantly upregulated in projected young tumor cells, whereas the normal old adult brain associated signature was significantly upregulated in projected old tumor cells (Fig. S1E), suggesting that ‘projected age’, as a computational metric, could reflect the extent of senescence age in tumor cells.

Since upregulated genes in the projected young tumor cells might act as inhibitors of senescence and genes specifically upregulated in tumor cells were prime candidates for biomarkers and therapeutic targets in the clinical application, we focused solely on genes with elevated expression level in tumor and further reduced the complexity of the senescence-related features based on multiple glioma cohorts (Fig. 1D). Firstly, we performed differential expression analysis between projected young and projected old tumor cells, normal and tumor cells in one glioma scRNA-seq as well as GBM and normal brain tissues, and identified 94 common genes that were highly expressed in both relatively young tumor cells and tumor tissues (Fig. 1E). The functions of these genes were closely related to cell mitosis, telomere elongation, DNA replication and damage, suggesting the important role of them in tumor malignancy (Fig. S1F). Notably, these genes were also involved in signaling pathways regulating cellular senescence and DNA repair (Fig. 1F), further demonstrating a close link between them and senescence. To improve the reliability of this gene set, we then intersected it with the DEGs between young and senescent brain cells from the aging mouse brain atlas (Fig. S1G). Ultimately, we identified 43 potential genes that might antagonize tumor cell senescence, which were highly expressed in young normal brain cells (Fig. 1G). We defined these genes as Projected Senescence Associated Signatures in Glioma (PSAG). To elucidate the biological programs encoded by PSAG, we performed functional enrichment analysis of these genes and found that the functions of PSAG could be broadly grouped into 8 classes, including cell cycle, cell migration, chromosome segregation, DNA damage checkpoint, microtubule formation, protein modification, response to stress and transcriptional regulation (Fig. S1H). Similarly, each gene in PSAG was also highly expressed in projected young tumor cells in another glioma scRNA-seq (Fig. 1H). Although most studies about senescence focused on normal cells, targeting cell senescence to prevent cancer cell proliferation and avoid the accumulation of genomic instability has become a new therapy for inhibiting tumor progression [12]. Therefore, further in-depth research on the biological mechanisms of PSAG-related core genes in the process of tumor cell senescence had important clinical application value.

### FAM83D in the PSAG may be a potential target for glioma treatment

**Fig. 2:**
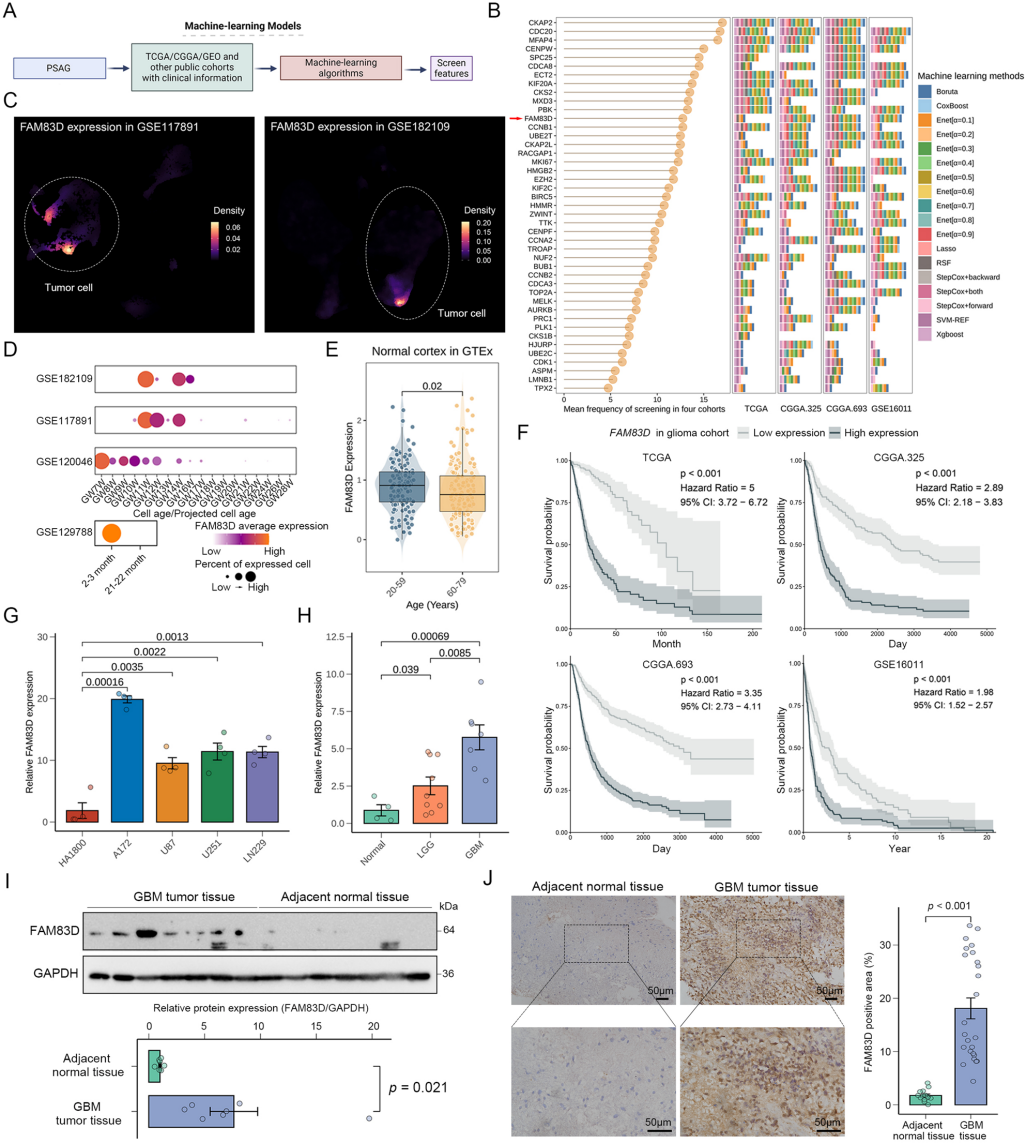
FAM83D in the PSAG may be a potential target for glioma. **A** Workflow for feature selection based on PSAG and the machine learning framework Mime. **B** Genes in PSAG with the frequency of screening by different machine learning models across four glioma cohorts. The bar chart on the right shows specific screening models of each gene in each cohort. The lollipop chart on the left shows the average frequency of screening in each gene. **C** Expression of *FAM83D* among different cell types in scRNA-seq. The white dashed circle represents the location of tumor cells. **D** Relationship between the expression of *FAM83D* and cell age in scRNA-seq. **E** Relationship between the expression of *FAM83D* and individual age in normal human cerebral cortical tissues. *p* value, two-sided unpaired Wilcoxon test. **F** Relationship between the expression of *FAM83D* and patient prognosis. *p* value, log-rank test. **G** Relative expression of *FAM83D* in different cell lines. Each data point represents one biological replicate (*n* = 4 replicates for each cell line). *p* value, two-sided unpaired t-test; error bars, mean ± SEM. **H** Relative expression of *FAM83D* in different glioma tissues. Each data point represents one biological replicate (*n* = 7 replicates for GBM, *n* = 9 replicates for LGG, *n* = 4 replicates for adjacent normal brain). *p* value, two-sided unpaired t-test; error bars, mean ± SEM. **I** Representative WB image of FAM83D in different tissues (top) and quantification of protein level normalized to the normal group (bottom). Each data point represents one biological sample (*n* = 7 biologically independent samples for each group). *p* value, two-sided unpaired t-test; error bars, mean ± SEM. **J** Representative IHC image of FAM83D in different tissues (left) and quantification of protein level normalized to the normal group (right). Each data point represents one random region of interest (*n* = 5 biologically independent samples for GBM, *n* = 3 biologically independent samples for adjacent normal brain, *n* = 5 random regions for each sample). *p* value, two-sided unpaired t-test; error bars, mean ± SEM

To identify potential targets for further research, we used a previously constructed machine-learning framework, Mime, to select core features (Fig. 2A). Among the top 15 most frequently selected genes (Fig. 2B), most exhibited elevated expression level in young adult brain, with *PBK*, *CKAP2L*, *CENPW*, *CCNB1*, *CDC20*, and *FAM83D* showing the most pronounced differences (Fig. S2A). These genes, except *FAM83D*, have been previously linked to senescence in various diseases [62–66]. In contrast, FAM83D is known to participate in cell cycle regulation and intracellular signal transduction by interacting with microtubule-associated proteins [18, 19]. Currently, this gene remains understudied in the context of senescence and tumor biology, with particularly limited prior investigation into its functional role in glioma. This gap in knowledge presents a significant opportunity for novel discovery, thus we select it for further exploration. At the single-cell level, *FAM83D* were major expressed in tumor cells suggesting a promising potential target of it which was specific to tumor cells in glioma TME (Fig. 2C). In addition, *FAM83D* expression was obviously elevated in the projected young glioma cells, normal brain cells at early developmental stages, young mouse brain cells and young normal cortex tissues (Fig. 2D, E). These data highlighted that FAM83D might be closely associated with the anti-senescence process in glioma.

We further investigated the relationship between *FAM83D* expression and other clinical characteristics in four independent glioma cohorts and found that high expression level of *FAM83D* was significantly associated with increased tumor grade, GBM histology, IDH wild-type status, 1p/19q non-co-deletion status, and enrichment of the mesenchymal (MES) subtype (Fig. S2B, C). Similar results were also detected in previously established glioma scRNA-seq (Fig. S2D). These correlations with known glioma-associated molecular biomarkers suggested that *FAM83D* expression increased with glioma malignancy. Given that *FAM83D* was highly expressed in high-grade gliomas and the poorly prognostic MES subtype, we next investigated the prognostic value of *FAM83D*. Of note, patients with elevated expression levels of *FAM83D* exhibited shorter overall survival across all glioma cohorts (Fig. 2F). Multivariate Cox regression analysis across multiple cohorts, adjusting by age, tumor grade, IDH mutation status, and MGMT methylation status, confirmed that *FAM83D* was an independent prognostic factor in glioma (Fig. S2E). To validate these findings, we collected various glioma cell lines and clinical glioma samples to evaluate the expression level of FAM83D. Consistently, the expression level of *FAM83D* was significantly elevated in tumor cells compared to normal astrocytes (Fig. 2G). Besides, the expression level of FAM83D in GBM tissues was significantly higher than that in adjacent normal brain tissues (Fig. 2H-J). Considering the important role of FAM83D in the cell cycle and the connection between abnormal cell cycle and cellular senescence, we hypothesized that glioma cells might resist cellular senescence by upregulating the expression of FAM83D.

### Knockdown of *FAM83D* induces tumor cell senescence to inhibit glioma progression

**Fig. 3:**
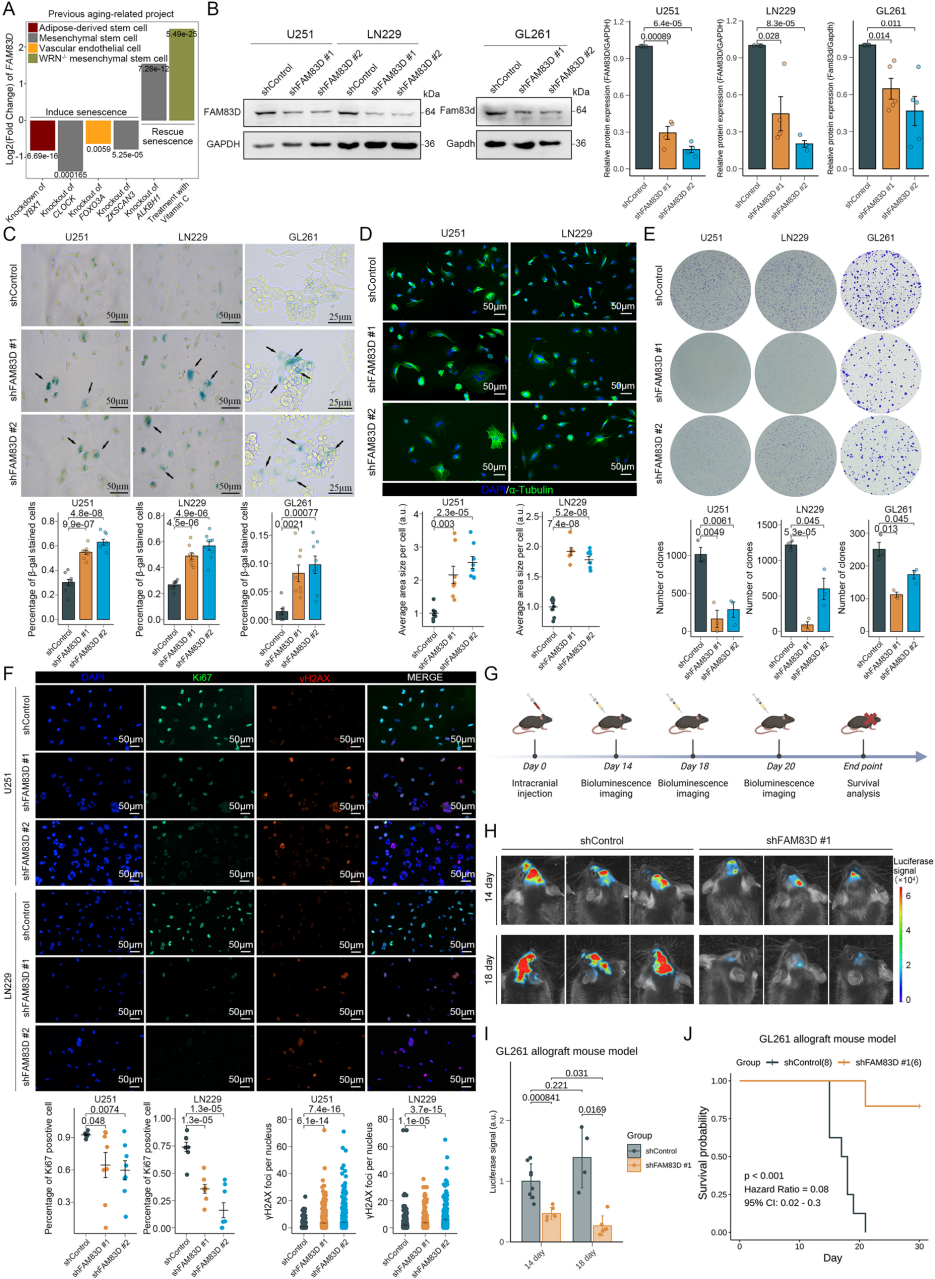
Knockdown of *FAM83D* induces glioma cell senescence. **A** Expression of *FAM83D* in different cells following senescence-induction in the Aging atlas. The number on the bar represents the corrected *p* value. **B** Representative WB image of FAM83D in U251, LN229, and GL261 cell lines under different conditions (left) and quantification of protein level normalized to the control group (right). Each data point represents one biological replicate (*n* = 4 replicates for U251 and LN229, *n* = 5 replicates for GL261). *p* value, two-sided unpaired t-test; error bars, mean ± SEM. **C** Representative staining image of SA-β-gal in U251, LN229 and GL261 cell lines under different conditions (top) and quantification of SA-β-gal positive cells normalized to the control group (bottom). Each data point represents one random region of interest (*n* = 9 random regions for each condition). *p* value, two-sided unpaired t-test; error bars, mean ± SEM. **D** Representative staining image of α-Tubulin in U251 and LN229 cell lines under different conditions (top) and quantification of cell sizes marked by α-Tubulin normalized to the control group (bottom). Each data point represents one random region of interest (*n* = 7–8 random regions for each condition). *p* value, two-sided unpaired t-test; error bars, mean ± SEM. **E** Representative colony formation in U251, LN229, and GL261 cell lines under different conditions (top) and quantification of colony number normalized to the control group (bottom). Each data point represents one biological replicate (*n* = 3 replicates for each condition). *p* value, two-sided unpaired t-test; error bars, mean ± SEM. **F** Representative staining image of Ki67 and γH2AX in U251 and LN229 cell lines under different conditions (top) and quantification of protein level normalized to the control group (bottom). Each data point represents one random region of interest (*n* = 8 random regions for each condition). *p* value, two-sided unpaired t-test; error bars, mean ± SEM. **G** Schematic of in vivo experiments using an allogeneic orthotopic glioma mouse model. **H** Representative bioluminescence image of mice on day 14 and day 18 under different conditions. **I** Quantification of luciferase signal in mice normalized to the control group on day 14 and day 18. Each data point represents one biological sample. *p* value, two-sided unpaired t-test; error bars, mean ± SEM. **J** Survival of mice in different conditions (*n* = 6 mice for shFAM83D, *n* = 8 mice for shControl). *p* value, log-rank test

To explore the relationship between the expression level of FAM83D and cellular senescence, we first investigated the transcriptomic alterations of *FAM83D* in different cell types following senescence-related induction in the Aging atlas. Consistent with our previous hypothesis, the expression level of *FAM83D* decreased in the senescent cells, while it increased in the young cells (Fig. 3A). We then selected two human glioma cell lines (U251 and LN229) and a mouse glioma cell line (GL261) for genetic manipulation. After lentivirus treatment, the transcription and protein levels of FAM83D were all significantly decreased in both knockdown groups (Fig. 3B and S3A). Meanwhile, the number of SA-β-gal positive tumor cells increased significantly after knocking down *FAM83D* (Fig. 3C). Cells in the knockdown group also increased in size, exhibiting pronounced atypia and multinucleation (Fig. 3D). These data suggested that knockdown of *FAM83D* could induce tumor cell senescence. Because the reliable detection of cellular senescence requires a combination of multiple techniques [67], we also evaluated the effects of knockdown of *FAM83D* on tumor cell proliferation and DNA damage, which are also the characteristics of cellular senescence. We observed that knockdown of *FAM83D* significantly inhibited the colony-forming ability of tumor cells (Fig. 3E). Furthermore, immunofluorescence analysis revealed a significant decrease of the expression of Ki67 and a significant increase of the expression of γH2AX in the knockdown group (Fig. 3F). Collectively, our findings supported that knockdown of *FAM83D* effectively inhibited tumor cell proliferation, promoted DNA damage and concurrently induced tumor cell senescence.

Subsequently, we used the GL261 cell line to establish an allogeneic orthotopic glioma mouse model for investigating the effect of knockdown of *FAM83D* on tumor progression in vivo (Fig. 3G). The intracranial tumors of mice in the control group gradually increased in size over time, while those in the knockdown group gradually decreased (Fig. 3H, I). In contrast to all mice harboring intracerebral GL261 on the control background, which started to succumb by day 14 post-modeling, some mice in the knockdown group were long-term survivors (Fig. 3J). These in vivo experiments suggested that tumor-derived FAM83D played an important role in glioma progression.

### Transcriptome sequencing uncovers abnormal mitosis in tumor cells affected by nuclear deletion of FAM83D

**Fig. 4:**
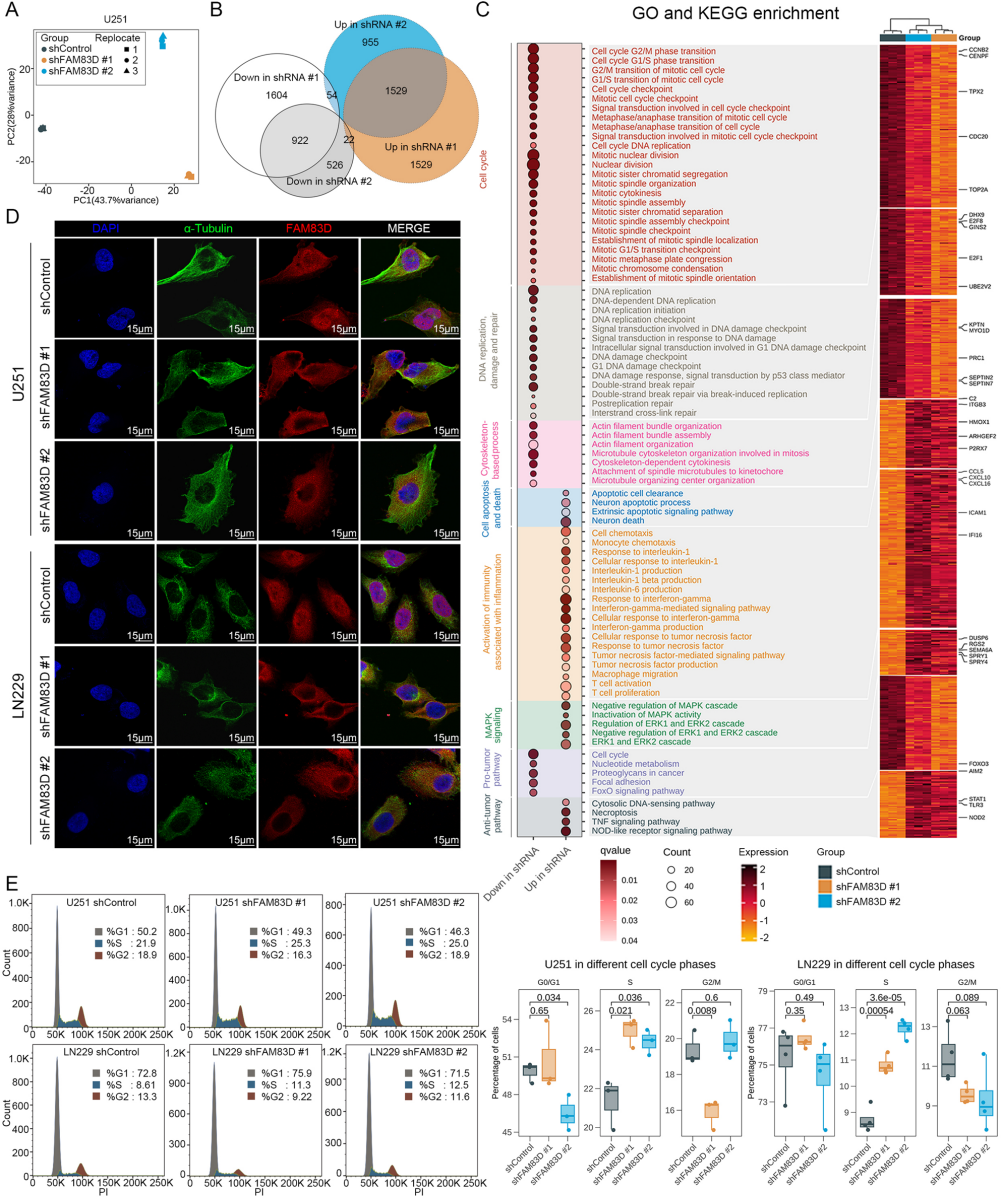
Transcriptome characteristics of tumor cells reprogrammed by knockdown of *FAM83D. ***A** PCA plot of the U251 cell line under different groups. Each data point represents one biological replicate (*n* = 3 replicates for each group). **B** Venn diagram showing DEGs between the knockdown group and the control group. **C** GO and KEGG enrichment based on common DEGs. The dot plot on the left represents the enrichment results of the corresponding biological processes and signaling pathways. The heatmap on the right corresponds to the expression of genes contained in different entries on the left in RNA-seq. q value, a corrected *p* value determined by the false discovery rate. **D** Representative staining image of α-Tubulin and FAM83D in U251 and LN229 cell lines under different conditions. **E** Representative flow cytometry image in U251 and LN229 cell lines under different conditions (left) and quantification of the percentage of each cell cycle phase (right). Each data point represents one biological replicate (*n* = 3 replicates for U251, *n* = 4 replicates for LN229). *p* value, two-sided unpaired t-test

To elucidate the involved molecular mechanisms, we performed transcriptome sequencing (RNA-seq) on U251 cells from different treatment groups. Preliminary quality control of the RNA-seq data revealed significant differences in transcriptome profiles between control and *FAM83D* knockdown groups (Fig. 4A and S3B). We compared the DEGs between these groups, and identified 1529 common genes with increased expression level and 922 common genes with decreased expression level in both knockdown groups (Fig. 4B). Functional enrichment analysis of these DEGs showed that knockdown of *FAM83D* significantly downregulated the expression of genes associated with cell cycle, DNA repair, and cytoskeleton assembly, while upregulated the expression of genes associated with apoptosis, activation of inflammatory immune activity, and negative regulation of MAPK signaling (Fig. 4C). Furthermore, pro-tumor pathways such as FoxO signaling pathway and tumor-related metabolic pathways were inhibited, while anti-tumor pathways such as cGAS-STING pathway, TNF signaling pathway, and necroptosis pathway were activated in the knockdown group (Fig. 4C). Alterations in the activity of these pathways might be the underlying mechanisms by which FAM83D regulated glioma cell senescence.

As cell cycle arrest was a key characteristic of senescent cells, and the interaction between FAM83D and microtubule-associated proteins played a key role in sister chromatid separation and equatorial plate assembly [18, 19], we hypothesized that knockdown of *FAM83D* might induce senescence by mediating abnormal cell division, a notion supported by the significant reduction in the expression level of genes related to spindle microtubules and centromere attachment in the knockdown group (Fig. 4C). By using confocal fluorescence microscopy, we observed co-localization of FAM83D and tubulin in the cytoplasm, indicating an interaction between these two proteins (Fig. 4D and S3C). Interestingly, the protein content of FAM83D in the nucleus was significantly reduced in the knockdown group (Fig. 4D and S3C). Additionally, after knockdown of *FAM83D* in tumor cells, the number of cells in the S phase was significantly increased, while the number of cells in the G0/G1 and G2/M phases tended to be decreased (Fig. 4E). These data suggested that knockdown of *FAM83D* could affect its nuclear localization leading to spindle positioning defects co-mediated by microtubules and FAM83D and thus impair normal cell cycle completion. 

### Knockdown of *FAM83D *increases extranuclear dsDNA to trigger tumor cell senescence by activating cGAS-STING signaling

**Fig. 5:**
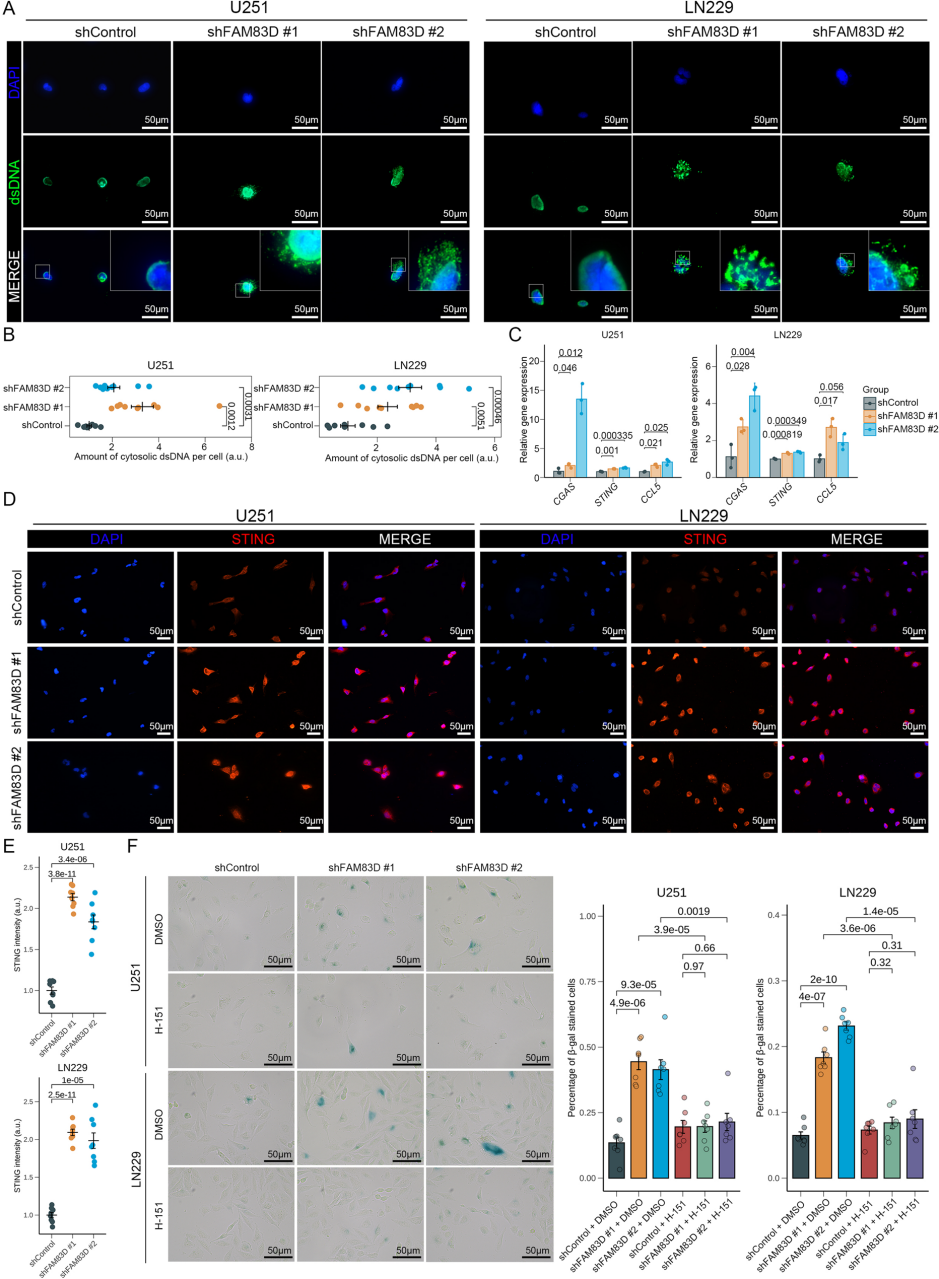
Activated cGAS-STING pathway involvement in tumor cell senescence induced by *FAM83D* knockdown. **A** Representative staining image of dsDNA in U251 and LN229 cell lines under different conditions. **B** Quantification of cytosolic dsDNA normalized to the control group based on (**A**). Each data point represents one random region of interest (*n* = 9 random regions for each condition). *p* value, two-sided unpaired t-test; error bars, mean ± SEM. **C** Relative expression of *CGAS*, *STING*, and *CCL5* in U251 and LN229 cell lines under different conditions. Each data point represents one biological replicate (*n* = 3 replicates for each condition). *p* value, two-sided unpaired t-test; error bars, mean ± SEM. **D** Representative staining image of STING in U251 and LN229 cell lines under different conditions. **E** Quantification of protein level normalized to the control group based on (**D**). Each data point represents one random region of interest (*n* = 8 random regions for each condition). *p* value, two-sided unpaired t-test; error bars, mean ± SEM. **F** Representative staining image of SA-β-gal in U251 and LN229 cell lines under different treatments (left) and quantification of SA-β-gal positive cells normalized to the control group (right). Each data point represents one random region of interest (*n* = 7 random regions for each treatment). Concentration of H-151 is 1 µM. *p* value, two-sided unpaired t-test; error bars, mean ± SEM

Abnormal cell division could lead to chromosomal instability, resulting in the accumulation of dsDNA in the cytoplasm, which then could be recognized by cGAS to activate downstream immune responses [68]. RNA-seq analysis of U251 cells also revealed the activation of the cGAS-STING signaling pathway in the knockdown group (Fig. 4C), suggesting its importance in the senescence phenotype induced by knockdown of *FAM83D*. In immunofluorescence staining, we found that the dsDNA content in the cytoplasm of the knockdown group increased significantly (Fig. 5A, B). Meanwhile, the expression level of genes associated with the cGAS-STING signaling pathway, such as *CGAS*, *STING*, and *CCL5*, was also significantly elevated after knockdown of *FAM83D* (Fig. 5C). We further verified the protein level of STING in different groups and found that the cytoplasmic STING in the knockdown group was also significantly increased (Fig. 5D, E). These data indicated that the cGAS-STING signaling pathway was dramatically activated in the tumor cells with the knockdown of *FAM83D*. To further determine the role of the cGAS-STING signaling pathway in tumor cell senescence induced by knockdown of *FAM83D*, we assessed the number of SA-β-gal positive cells and the expression of downstream senescence markers p16, p21 and NF-κB (p65) by adding the STING inhibitor H-151 to the cell culture medium. The results showed that the STING inhibitor significantly ameliorated the senescence phenotype induced by knockdown of *FAM83D* in tumor cells (Fig. 5F). In addition, knockdown of *FAM83D* comprehensively elevated the protein level of p16, p21 and phospho-p65 (p-p65) in tumor cells (Fig. S3D). Whereas, the STING inhibitor significantly reduced the protein level of these senescence markers induced by knockdown of FAM83D in tumor cells (Fig. S3D), which further confirmed the causal relationship between them.

### SASP factors induced by the knockdown of *FAM83D* reshape the tumor microenvironment and promote the senescence of neighboring tumor cells.

**Fig. 6:**
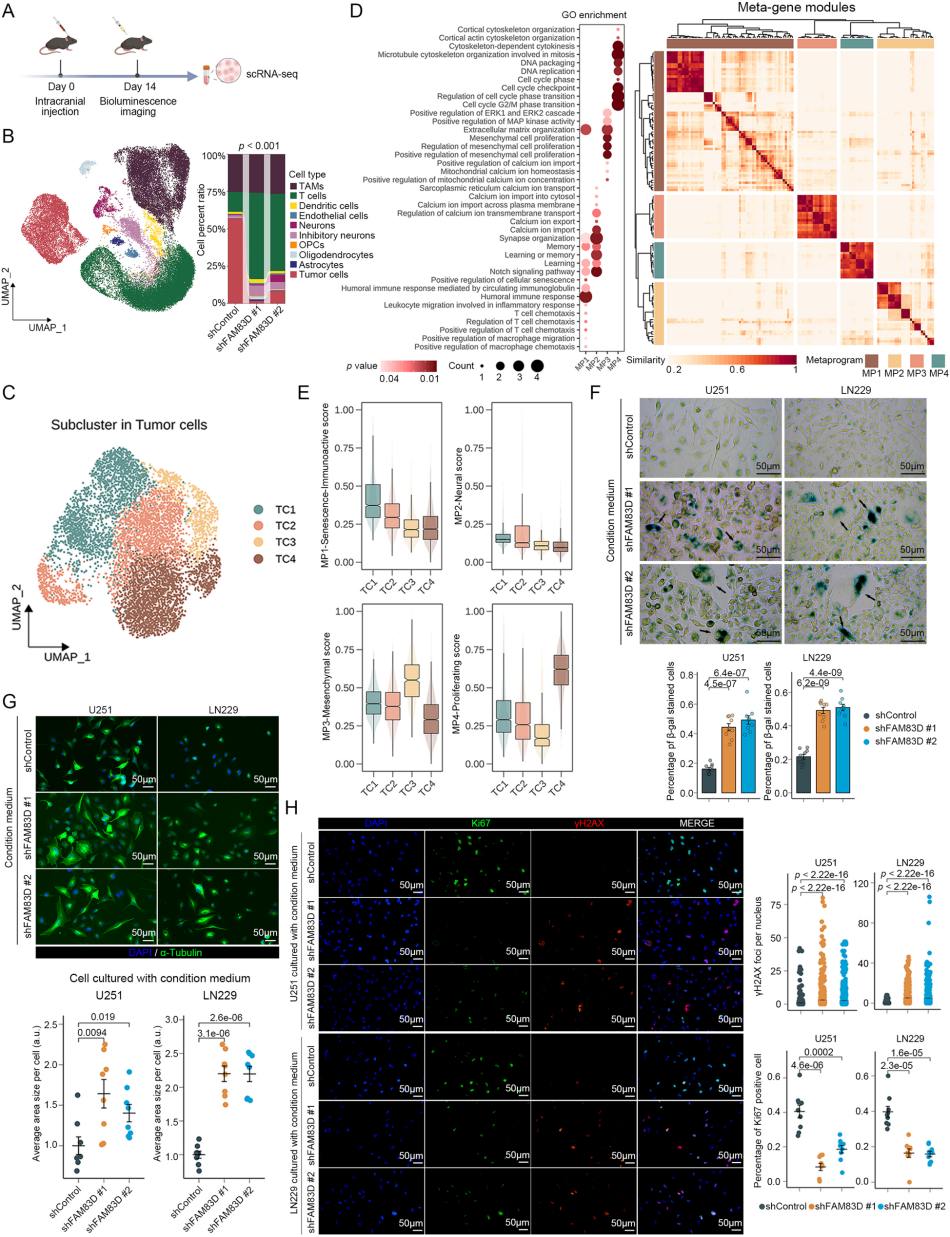
Knockdown of *FAM83D* reshapes the tumor microenvironment. **A** Workflow for scRNA-seq processing using an allogeneic orthotopic glioma mouse model. **B** UMAP plot of cells from scRNA-seq colored by cell types (left) and proportion of each cell type across samples, respectively (right). *p* value, Chi-square test. **C** UMAP plot of tumor cells from scRNA-seq colored by sub-clusters. **D** Similarity of MPs in tumor cell characteristics identified by the NMF algorithm (right) and GO biological process enrichment based on genes in each MP (left). The similarity is calculated using cosine similarity. **E** Enrichment scores of each MP in different tumor cell sub-clusters. **F** Representative staining image of SA-β-gal in wild-type U251 and LN229 cell lines treated with different conditioned medium (top) and quantification of SA-β-gal positive cells normalized to the control group (bottom). Each data point represents one random region of interest (*n* = 9 random regions for each treatment). The culture time is 48 h. *p* value, two-sided unpaired t-test; error bars, mean ± SEM. **G** Representative staining image of α-Tubulin in wild-type U251 and LN229 cell lines treated with different conditioned medium (top) and quantification of cell sizes marked by α-Tubulin normalized to the control group (bottom). Each data point represents one random region of interest (*n* = 8 random regions for each treatment). The culture time is 48 h. *p* value, two-sided unpaired t-test; error bars, mean ± SEM. **H** Representative staining image of Ki67 and γH2AX in wild-type U251 and LN229 cell lines treated with different conditioned medium (left) and quantification of protein level normalized to the control group (right). Each data point represents one random region of interest (*n* = 9 random regions for each condition). The culture time is 48 h. *p* value, two-sided unpaired t-test; error bars, mean ± SEM

In our RNA-seq of U251, we found significant alterations in the expression level of senescence-related genes in the knockdown group, including several SASP-related genes such as *IL1A*, *IL6*, *CCL26*, *TNFRSF11B*, and *GDF15* (Fig. S3E). This suggested that knockdown of *FAM83D* might induce a specific expression profile of SASP in tumor cells. To further analyze the impact of tumor-derived SASP induced by knockdown of *FAM83D* on the glioma TME, we performed scRNA-seq of the tumor sample from GL261 glioma–bearing mice on day 14, since the previously established mice in the knockdown group began to die on day 14 after modeling (Fig. 6A). After rigorous quality control, a total of 41,578 cells were obtained from brain tumor tissues across three mouse groups (Fig. S3F). Based on typical cell markers, these cells were classified into 10 major populations, including tumor-associated macrophages (TAMs, expressing *Ptprc*, *P2ry12*, and *Tmem119*), T cells (expressing *Ptprc*, *Nkg7*, and *Cd3d*), dendritic cells (expressing *Ptprc*, *Ccr7*, and *H2-Eb1*), endothelial cells (expressing *Cd34*, *Pecam1*, and *Vwf*), neurons (expressing *Trpm3*, *Pcsk2*, and *Atp2b3*), inhibitory neurons (expressing *Rbfox3*, *Nrxn3*, and *Gad1*), oligodendrocyte precursor cells (OPCs, expressing *Pdgfra*, *Olig2*, and *Sox2*), oligodendrocytes (expressing *Mog*, *Mag*, and *Olig1*), astrocytes (expressing *Gfap*, *S100b*, and *Slc4a4*) and tumor cells (expressing *Mki67*, *Top2a*, and *Nes*) (Fig. 6B and S3G). Combined with inferred copy number variations (CNV), tumor cells and other normal cells could be clearly distinguished (Fig. S3H). Notably, the number of tumor cells decreased in both knockdown groups, while the number of T cells and inhibitory neurons increased in both knockdown groups (Fig. 6B). This different distribution of cell types in the TME suggested that knockdown of *FAM83D* in the tumor cells reshaped the glioma microenvironment and might influence the functional status of surrounding cells.

To investigate tumor cell heterogeneity, we re-clustered tumor cells individually and identified 4 tumor sub-clusters (TC1-4) (Fig. 6C). Based on NMF algorithm, we also identified 4 meta-programs (MPs) within the tumor cell characteristics reflecting distinct cellular functions and states (Fig. 6D). Biological function enrichment results showed that features in MP1 were closely related to the regulation of immune cell function and cell senescence, and thus were defined as the senescence-mediated immune activation program (MP1-Senescence-Immunoactive); features in MP2 were closely related to neuronal electrical activity and ion transport, and thus were defined as the neural program (MP2-Neural); features in MP3 were closely related to mesenchymal cell activity, and thus were defined as the mesenchymal program (MP3-Mesenchymal); and features in MP4 were closely related to cell division and DNA replication, and thus were defined as the proliferating program (MP4-Proliferating) (Fig. 6D). By comparing the enrichment scores of these four MPs in different tumor cell sub-clusters, we noticed that the TC1 sub-cluster was mainly composed of senescence-immunoactive tumor cells (TC1: Sen-Imm-active-like); the TC2 sub-cluster was mainly composed of mixed-neural-mesenchymal tumor cells (TC2: Neu-Mes Mix-like); the TC3 sub-cluster was mainly composed of mesenchymal tumor cells (TC3: Mesenchymal-like); and the TC4 sub-cluster was mainly composed of proliferating tumor cells (TC4: Proliferating-like) (Fig. 6E). Consistent with the cell status defined by MPs, most cancer-associated pathways were activated in the TC3, highlighting its more malignant status, while interferon-associated pathways were activated in the TC1, highlighting its ability to activate immune cells (Fig. S4A). Of note, TC1 was predominantly enriched in the knockdown group, whereas TC3 was specifically enriched in the control group, indicating that senescence-mediated immune activation in TC1 induced by knockdown of *FAM83D* was a key factor in remodeling TME (Fig. S4B). We further evaluated the activity of cellular senescence and the cGAS-STING signaling pathway in different sub-clusters and found that the cGAS-STING signaling was significantly activated in knockdown groups across all sub-clusters (Fig. S4C). Meanwhile, the cellular senescence was also tended to be activated in knockdown groups across all sub-clusters (Fig. S4C). Therefore, we hypothesized that senescent tumor cells induced by knockdown of *FAM83D* via activation of cGAS-STING signaling might secrete SASP factors to promote the senescence of neighboring non-senescent tumor cells and stimulate immune cell activity in TME for suppressing glioma progression.

Then, we collected the conditioned medium from tumor cells under different treatments to culture wild-type tumor cells for investigating the function of SASP factors induced by knockdown of *FAM83D*. Interestingly, the conditioned medium from knockdown groups significantly promoted the senescence of wild-type tumor cells with increased cell size and pleomorphism (Fig. 6F, G). In addition, the conditioned medium from knockdown groups also significantly inhibited the proliferation but increased the DNA damage of wild-type tumor cells (Fig. 6H). These data confirmed our hypothesis about the effect of tumor-derived SASP factors induced by knockdown of *FAM83D* on neighboring tumor cells.

### Knockdown of *FAM83D *in tumor cells drives macrophage polarization toward the M1 state

Given the observed alterations in immune cell numbers within our scRNA-seq dataset, we sought to investigate whether knockdown of *FAM83D* in tumor cells could modulate immune cell functions. We first re-clustered T cells and identified 4 clusters, including CD8 T cells (expressing *Cd8a*, *Cd8b1*, and *Gzmk*), CD4 T cells (expressing *Cd4*, *Tcf7*, and *Tnfrsf18*), Natural Killer T cells (NK T cells, expressing *Nkg7*, *Klrd1*, and *Gzma*), and proliferating T cells (expressing *Mki67*, *Top2a*, and *Cdk1*) (Fig. S4D, E). Notably, the number of CD8 T cells increased in both knockdown groups, whereas the number of CD4 T cells altered slightly (Fig. S4F). Examining T-cell functional states revealed that the cytotoxic activity of CD8 T cells was significantly elevated upon the knockdown of *FAM83D*, while their exhausted states was significantly reduced (Fig. S4G). In addition, the regulatory state of CD4 T cells, as an immunosuppressive state, was also decreased in the knockdown group (Fig. S4H), suggesting a shift toward a more immunoactive microenvironment induced by the knockdown of *FAM83D*.

**Fig. 7:**
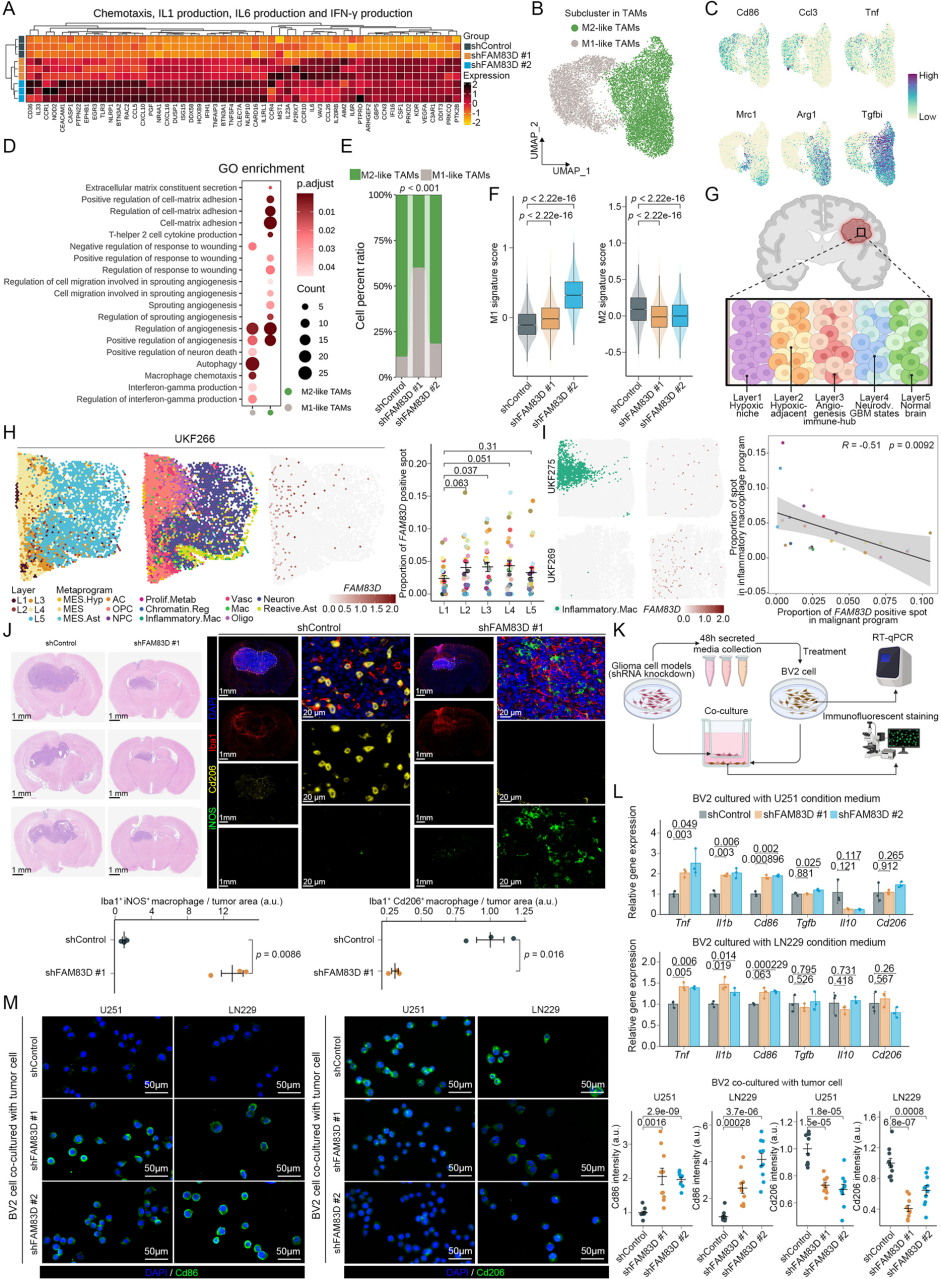
Knockdown of *FAM83D* leads to the infiltration of M1 TAMs. **A** Expression of genes associated with chemotaxis, IL1 production, IL6 production, and IFN-γ production in RNA-seq of the U251 cell line. **B** UMAP plot of TAMs from scRNA-seq colored by sub-clusters. **C** Expression of M1 markers (*Cd86*, *Ccl3*, and *Tnf*) and M2 markers (*Mrc1*, *Arg1*, and *Tgfbi*) in TAMs from scRNA-seq. **D** GO biological process enrichment based on the corresponding marker genes in each TAMs sub-cluster. *p* adjusted value, a corrected *p* value determined by bonferroni. **E** Proportion of each TAM’s sub-cluster across samples, respectively. *p* value, Chi-square test. **F** Enrichment scores of M1/M2 macrophage associated signatures in TAMs across samples. *p* value, two-sided unpaired Wilcoxon test. **G** Spatial structure model of glioma based on spatial transcriptomics. **H** Representative sample UKF266 showing the expression of *FAM83D* in each layer (left) and quantification of *FAM83D* positive spots in each layer (right). *p* value, two-sided unpaired t-test; error bars, mean ± SEM. **I** Representative samples UKF269 and UKF275 showing the relationship between the expression of *FAM83D* and the proportion of inflammatory macrophage (left) and their correlation analysis (right). *p* value, Pearson’s correlation test. **J** Representative HE staining image and staining image of Iba1, iNOS, and Cd206 in mouse brains under different treatments (top) and quantification of protein level normalized to the control group (bottom). Each data point represents one mouse (*n* = 3 mice for each treatment). *p* value, two-sided unpaired t-test; error bars, mean ± SEM. **K** Schematic of cellular experiments verifying the effect of SASP induced by knockdown of *FAM83D* on macrophage polarization. **L** Relative expression of M1 markers (*Tnf*, *Il1b*, and *Cd86*) and M2 markers (*Tgfb, Il10*, and *Cd206*) in the BV2 cell line treated with different conditioned medium. Each data point represents one biological replicate (*n* = 3 replicates for each treatment). The culture time is 48 h. *p* value, two-sided unpaired t-test; error bars, mean ± SEM. **M** Representative staining image of Cd86 and Cd206 in the BV2 cell line co-cultured with tumor cells under different conditions (left) and quantification of protein level normalized to the control group (right). Each data point represents one random region of interest (*n* = 10 random regions for each condition). The culture time is 48 h. *p* value, two-sided unpaired t-test; error bars, mean ± SEM

Since TC1 exhibited immune activation characteristics and genes related to macrophage polarization, including chemokines, IL-1, IL-6, and IFN production were significantly upregulated in U251 with knockdown of *FAM83D* (Fig. 7A), we next focused on TAMs, which played a crucial role in glioma TME. Although the proportion of TAMs in knockdown groups increased only slightly (Fig. 6B), their functional states might differ. We performed de novo re-clustering in pooled TAMs and identified two sub-clusters, which expressed classic M1/M2 markers and were defined as M1-like TAMs (expressing *Cd86*, *Ccl3*, and *Tnf*) and M2-like TAMs (expressing *Mrc1*, *Arg1*, and *Tgfbi*) (Fig. 7B, C). Biological function enrichment revealed that the characteristics of M1-like TAMs were associated with interferon signaling and autophagy, while the characteristics of M2-like TAMs were associated with angiogenesis, tissue repair, and cell-extracellular matrix adhesion, consistent with the functions of their respective type (Fig. 7D). Notably, M1-like TAMs was predominantly enriched in the knockdown group, whereas M2-like TAMs was specifically enriched in the control group, indicating that knockdown of *FAM83D* in tumor cells might drive the polarization of TAMs in TME (Fig. 7E). Overall, the expression level of M1 macrophage-related features were also significantly increased in the knockdown group, while it was opposite for M2 macrophage-related features, further confirming our hypothesis (Fig. 7F).

A previous study had constructed a glioma spatial model dominated by a hypoxia gradient containing 5 layers of adjacent structures with infiltration of different cell types and states defined by 6 non-malignant MPs and 8 malignant MPs (Fig. 7G and S5A-C). In this model, tumor cells and macrophages were mainly located in layer 2 (L2) to layer 4 (L4) (Fig. S5A-C). Intriguingly, we noticed that *FAM83D* was predominantly expressed in L2 to L4 (Fig. 7H). These three layers were spatially adjacent to each other, suggesting that *FAM83D*-expressing tumor cells and macrophages might be spatially close, facilitating the paracrine effects of tumor-derived SASP factors on macrophages. Besides, we also found that a higher abundance of *FAM83D*-expressing tumor cells was significantly correlated with a lower abundance of inflammatory macrophages, while no such correlation was observed with non-inflammatory macrophages (Fig. 7I and S5D). To verify these findings, we quantified TAMs infiltration in the glioma TME on day 14 post-implantation using canonical M1/M2 macrophage markers in tumor-bearing mouse brain tissue. Compared with the control group, Cd206-positive TAMs were depleted in *FAM83D*-knockdown tumors, whereas iNOS-positive TAMs were enriched (Fig. 7J). Therefore, we speculated that tumor cells with knockdown of *FAM83D* could secrete specific SASP factors to promote macrophage polarization toward the M1 phenotype. Similarly, we collected the conditioned medium from tumor cells under different treatments to culture microglia or used a co-culture model of tumor cells and microglia in vitro to mimic the effects of tumor-derived SASP factors induced by knockdown of *FAM83D* on TAMs (Fig. 7K). Indeed, microglia cultured with conditioned medium from the knockdown group showed significantly increased expression level of M1 macrophage markers (*Tnf*, *Il1b*, and *Cd86*) at the transcriptional level, while the expression level of M2 macrophage markers (*Tgfb*, *Il10*, and *Cd206*) remained unchanged (Fig. 7L). In the co-culture model, microglia cultured with tumor cells with knockdown of *FAM83D* exhibited a marked increase of Cd86 in protein level but a concomitant decrease of Cd206 (Fig. 7M). These results were consistent with our findings above.

### ANXA1-FPR1/2 ligand-receptor signaling involved in tumor cells-TAMs crosstalk is associated with macrophage polarization

**Fig. 8:**
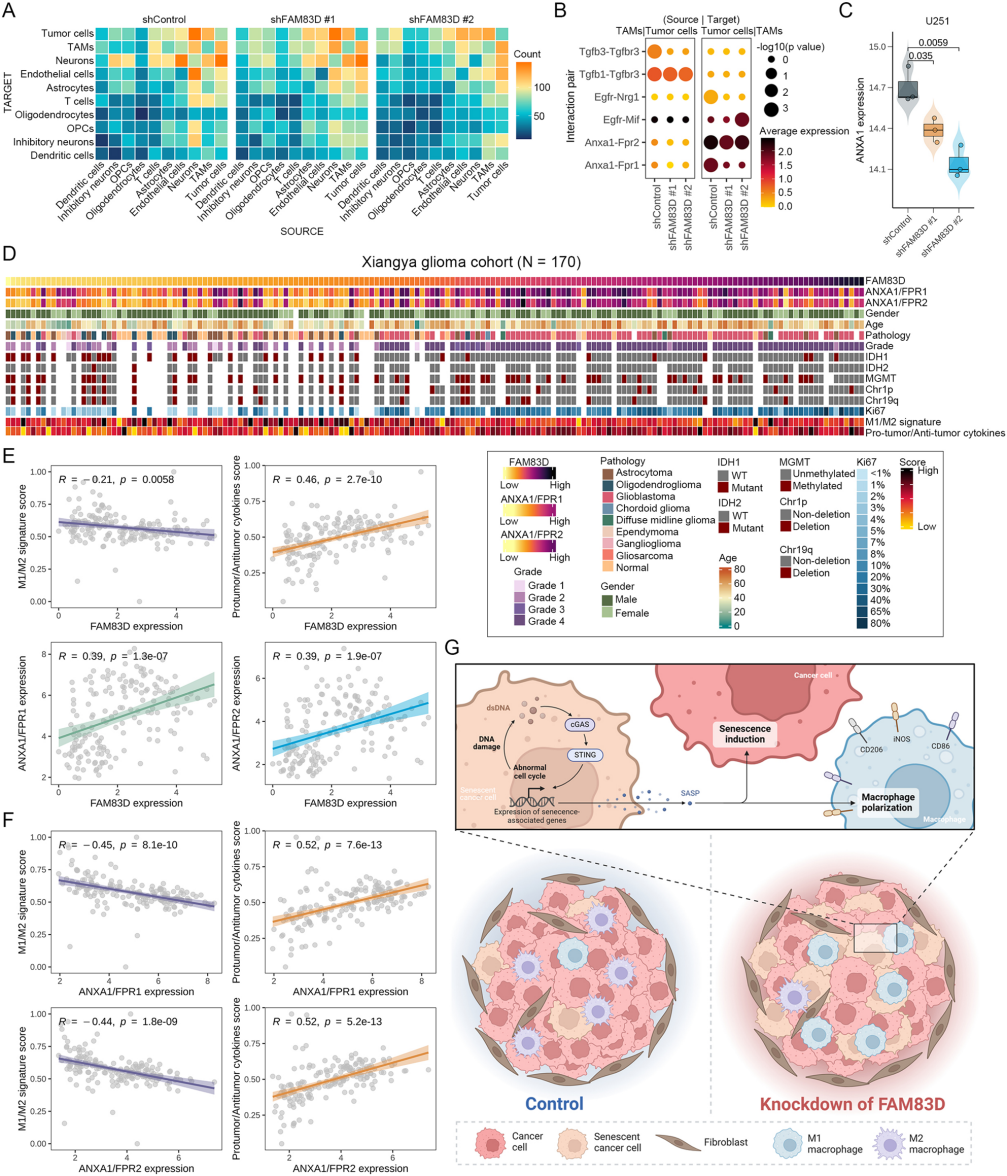
ANXA1-FPR1/2 signaling is associated with the polarization of TAMs regulated by FAM83D in glioma. **A** Intercellular communication networks in scRNA-seq. **B** Specific ligand-receptor signals between tumor cells and TAMs in scRNA-seq. **C** Expression of *ANXA1* in RNA-seq of the U251 cell line. *p* value, two-sided unpaired Wilcoxon test. **D** Heatmap showing the relationship between the expression of *FAM83D* and other clinical characteristics in an independent glioma cohort (Xiangya glioma cohort). ANXA1-FPR1/2, the average expression of *ANXA1* and *FPR1/2*; M1/M2 signature, the enrichment ratio of M1 macrophage-associated signatures to M2 macrophage-associated signatures; Pro-tumor/Anti-tumor cytokines, the enrichment ratio of pro-tumor to anti-tumor cytokines. **E** Correlation analysis between the expression of *FAM83D* and other signatures based on (**D**). *p* value, Pearson’s correlation test. **F** Correlation analysis between the ANXA1-FPR1/2 signaling and other signatures based on (**D**). *p* value, Pearson’s correlation test. **G** Summary of FAM83D in regulating glioma cell senescence and shaping tumor microenvironment with a specific impact on macrophage polarization

As the SASP factors could mediate intercellular communication, a comparative analysis of intercellular communication networks across groups based on scRNA-seq might reveal key factors driving macrophage polarization. In our scRNA-seq, intercellular communication networks within the TME, regardless of control or knockdown group, were primarily concentrated between tumor cells, neurons, TAMs and endothelial cells (Fig. 8A). We further focused on specific ligand-receptor signaling between tumor cells and TAMs and identified several ligand-receptor pairs that exhibited differential communication between control and knockdown groups (Fig. 8B). For example, Egfr-Mif ligand-receptor signaling was increased in the knockdown group, while Egfr-Nrg1 and Tgfb3-Tgfbr3 ligand-receptor signaling were increased in the control group (Fig. 8B). TGF-β and EGFR signaling were both known to promote tumor growth, and macrophage migration inhibitory factor (MIF) had been reported to compete with EGF for binding to EGFR, thereby inhibiting the activation of EGFR signaling [69]. Among these networks, we noticed that the signaling of two ligand-receptor pairs associated with Anxa1 was significantly reduced between tumor cells and TAMs in the knockdown group (Fig. 8B). It had been reported that ANXA1 could be secreted into the extracellular environment and regulate cell behavior in a paracrine manner by activating formyl peptide receptors 1 and 2 (FPR1/2) [70]. There were also other studies reporting that ANXA1 was closely related to cell senescence and SASP, and could inhibit the anti-tumor activity of immune cells by inducing the production of M2 macrophages through activating FPR1 [71, 72]. These data suggested that tumor cells with knockdown of *FAM83D* might regulate macrophage polarization by reducing the level of ANXA1 in the SASP. Indeed, the expression level of ANXA1 was significantly downregulated in tumor cells after knockdown of *FAM83D* (Fig. 8C).

To expand our findings to clinical applications, we evaluated the correlation between the expression level of *FAM83D*, ANXA1-FPR1/2 ligand-receptor signaling, M1/M2 macrophage-associated signatures, and immune-related cytokines in a clinical cohort of gliomas encompassing virtually all known pathological types. In 170 glioma clinical samples, the expression level of *FAM83D* and ANXA1-FPR1/2 ligand-receptor signaling was elevated with increased glioma malignancy (Fig. 8D and S5E, F). Meanwhile, the expression level of *FAM83D* was significantly positively correlated with ANXA1-FPR1 signaling, ANXA1-FPR2 signaling, and the enrichment ratio of pro-tumor to anti-tumor cytokines, whereas being significantly negatively correlated with the enrichment ratio of M1 macrophage-associated signatures to M2 macrophage-associated signatures (Fig. 8E). Similarly, both ANXA1-FPR1 and ANXA1-FPR2 signaling were significantly positively correlated with the enrichment ratio of pro-tumor to anti-tumor cytokines, and significantly negatively correlated with the enrichment ratio of M1 macrophage-associated signatures to M2 macrophage-associated signatures (Fig. 8F). Taken together, these results not only supported our hypothesis, but also paved the way for the clinical applications of these markers to assess tumor progression and as potential therapeutic targets.

## Discussion

Single-cell RNA sequencing has opened up new avenues for studying the complex cancer ecosystem and providing an unprecedented opportunity to decipher the tumor heterogeneity, which has been widely used to identify new cell types involved in tumor progression and treatment resistance [73]. In our study, we proposed a novel concept of age heterogeneity in tumor cells and developed a new algorithm, CellToAge, to identify the relative age of tumor cells based on scRNA-seq. We found that the mapped developmental age of each tumor cell within the same sample was variable and did not correspond to the actual aging level of the patient. Therefore, simply using the patient’s actual age as the age of tumor cells for subsequent analysis would significantly impact our understanding of senescence-related characteristics in tumor cells. Due to that PSAG was constructed based on the glioma datasets, we consider that PSAG represents a glioma-specific anti-senescence program. However, in PSAG, some genes have been universally confirmed to participate in the regulation of cellular senescence in various diseases. For example, the deficiency of CDK1, a central regulator driving cells through the G2 phase, can lead to the arrest of proliferation and premature cellular senescence in mouse embryonic fibroblasts [74]; inhibition of PLK1 can lead to delayed mitotic entry and spindle abnormalities in some cancer cell lines, thereby inducing cellular senescence [75]; mice with *HMGB2* deficiency exhibit earlier onset of severe senescence-related cartilage surface damage [76]; and inhibition of EZH2 can stimulate the production of inflammatory SASP factors to promote the infiltration of natural killer cells and T cells, thereby enhancing in vivo immune surveillance of pancreatic tumors [77]. The basic cellular functions mediated by these genes may reflect PSAG as an evolutionarily conserved molecular characteristic for anti-senescence. Therefore, PSAG is very likely to be a general anti-senescence program, but it still needs to be further verified through different disease models.

Cell cycle is a demanding process for normal cells, involving a series of ordered events to ensure the proper replication and separation of the genome. Two key events are included in this process: the DNA replication phase (S phase), in which the nuclear genome replicates; and the mitotic phase (M phase), in which chromosomes condense, sort, and distribute evenly to daughter cells [78]. Many studies have shown that cells in the S and M phases are susceptible to external disturbances, resulting in abnormalities. Therefore, cells during this period are monitored and protected by a series of cell cycle checkpoints, including DNA damage, DNA replication, and spindle assembly checkpoints, to ensure the completion of the cell cycle [79]. Among them, the spindle assembly checkpoint monitors whether each chromosome is attached to the mitotic spindle through the centromere located on the equatorial plate to ensure accurate chromosome segregation [80]. Incorrect spindle positioning and abnormal spindle function can cause cells to erroneously segregate a small number of chromosomes in one or more divisions, ultimately resulting in chromosomal instability [80, 81]. Excessive accumulation of chromosomal instability can lead to cells entering a brief quiescent or senescent state, or even apoptosis. FAM83D recruits CK1α (a member of the casein kinase family) and Kid (a member of the chromokinesin family) to co-localize on the spindle during mitosis, playing a crucial regulatory role in ensuring proper spindle localization and the formation of the equatorial plate [18, 19]. In this study, knockdown of *FAM83D* reduced the localization of FAM83D in the nucleus, which might severely affect the normal function of the spindle during cell division, leading to abnormal cell cycle progression. We also observed cellular and nuclear atypia in tumor cells with knockdown of *FAM83D*, both related to abnormal cell cycles and contributing to cellular senescence.

Increasing evidence suggests that the cGAS-STING signaling pathway is essential for the regulation of cellular senescence and cancer progression [82]. ​​A key clue regarding the regulatory role of the cGAS-STING signaling pathway in cellular senescence comes from a study of cGAS^−/−^ mouse embryonic fibroblasts, which transformed into immortalized cells more quickly in vitro than wild-type embryonic fibroblasts [82, 83]. Mechanistic investigations indicate that this is because cGAS senses DNA damage, promoting the expression of p16 and SA-β-gal, thereby inducing senescence [83]. Furthermore, during mitosis, misseparation of chromosomes can lead to delayed chromosomes that are excluded from the main nucleus and subsequently become enclosed by their own membrane, thereby forming micronuclei in the cytoplasm [68]. Micronucleus membranes are easily disrupted, and once ruptured, the released dsDNA can be recognized by cGAS to trigger downstream signals [84, 85]. Given the accumulation of cytoplasmic dsDNA and the crucial role of DNA damage signaling in cellular senescence, it is not difficult to establish a link between the cGAS-STING signaling pathway and cellular senescence. In our study, we likewise noticed that knockdown of *FAM83D* increased nuclear DNA damage and cytoplasmic dsDNA, and activated the cGAS-STING signaling pathway. Conversely, inhibition of STING alleviated the senescent phenotype induced by knockdown of *FAM83D*, highlighting the important role of the cGAS-STING signaling pathway in FAM83D-regulated glioma cell senescence. Of course, further validation of the expression of micronuclei and downstream signaling molecules of cGAS-STING at different molecular levels would greatly enhance the reliability of our findings.

It is also important to note that cellular senescence is a multifaceted process orchestrated by a network of pathways, thus the induction of senescence may be not fully dependent on cGAS-STING pathway. In fact, the DNA damage likely acts upstream of cGAS-STING in many scenarios by generating cytoplasmic DNA fragments that serve as cGAS ligands [86]. The interferons and inflammatory factors produced downstream by cGAS-STING can in turn affect the activity or stability of the DNA damage pathway [86]. Specifically, DNA damage can activate the p53-p21 and p16-Rb pathways to block the cell cycle and implement senescence programs [86]. While, in our glioma model, the inhibition of cGAS-STING pathway reversed the protein level of p16 and p21 in senescent tumor cells, highlighting the closely connection between cGAS-STING pathway and DNA damage pathway in mediating tumor cell senescence upon the knockdown of *FAM83D*. Additionally, the relative contribution of these pathways appears to be highly context-dependent and varying with the cell types [87]. For instance, the senescence of hematopoietic stem cells may be more dependent on p16 or mitochondrial dysfunction, while the role of cGAS-STING pathway is not yet clear [88]. Future studies comparing different cell types, especially the subtypes of glioma cells, will be essential to map the hierarchical activation of these parallel and interacting pathways in defining the senescent phenotype.

SASP, a unique characteristic of senescent cells, can enhance their own senescence through autocrine signaling and also influence the tissue microenvironment by transducing paracrine signals to neighboring tumor cells and non-tumor cells [89–91]. While, our data revealed an anti-tumor SASP profile triggered by knockdown of *FAM83D* in glioma. The SASP factors upregulated in our model, such as IL1A, IL6, and CXCL10, are potent chemoattractants for anti-tumor immune cells and inducers of M1 macrophage polarization. In contrast, the pro-tumorigenic SASP factors for cell migration and growth stimulatory, such as IL8, HGF, and MMPs, do not show obvious alteration. This divergence likely stems from the unique activation of cGAS-STING signaling pathway, which has been reported to induce the specific SASP profile to promote the infiltration of immune cells, such as cytotoxic T cells and M1 TAMs, into tumor areas in cancer, thereby enhancing anti-tumor immunity [92, 93]. Currently, therapy-induced senescence has been used as an initial anti-tumor treatment, in which senescent tumor cells can further promote tumor suppression by inhibiting the activity of neighboring tumor cells, improving intravascular drug delivery, and recruiting immune cells [12, 94]. Our data on cell and mouse models revealed that tumor-derived SASP induced by knockdown of *FAM83D* not only promoted the senescence of neighboring tumor cells, but also facilitated M1 polarization of macrophages to activate anti-tumor immune responses, which might be the reason for the long-term survival of mice in the knockdown group. Through cell-cell communication analysis, we noticed a ligand, ANXA1, that could function as both a membrane protein and a secretory protein [70], whose associated signaling was significantly reduced in the knockdown group. ANXA1 is an immunomodulatory protein that has been shown to be overexpressed in a variety of cancer types and to promote cancer progression [95]. It has been reported that endothelial cells with knockdown of *FAM83D* exhibit a senescence phenotype and express some inflammation-associated SASP factors [72]. Recently, a glioma clinical trial (NCT03392545) demonstrated that ANXA1 can induce the production of M2 macrophages and microglia through interaction with its receptor FPR1, establishing a Treg cell-driven immunosuppressive microenvironment [71]. In addition, a humanized IgG1 monoclonal antibody, MDX-124, has been reported to specifically bind to ANXA1 in human cancer cell lines, disrupting its interaction with FPR1/2 to inhibit the proliferation of tumor cells [95]. Combined with the analysis in our independent glioma cohort, we consider that knockdown of *FAM83D* may regulate macrophage polarization by reducing the level of ANXA1 in the SASP.

Sustained treatment-induced senescence can also be harmful, as the accumulation of senescent cells reaching a certain threshold in the body can produce systemic effects and cause various senescence-related diseases [96]. In addition, SASP can also promote angiogenesis and epithelial-mesenchymal transition of tumor cells, thereby enhancing the migration and metastasis of tumor cells [12]. For example, radiotherapy-induced senescent astrocytes can activate MET in GBM by secreting SASP factors, such as hepatocyte growth factor, thereby promoting the invasiveness and recurrence of GBM [97]. Another GBM study reveals that clearing malignant senescent cells expressing p16^INK4A^, which account for less than 7% of the tumor, can alter the tumor ecosystem and improve the survival rate of tumor-bearing mice [98]. It can be seen that induced cellular senescence can inhibit tumors in the short term, while residual senescent cells that are not cleared in the long term may promote tumor recurrence [94]. Therefore, balancing the “bright side” and the “dark side” of senescence is crucial for optimizing pro-senescence therapy in cancer.

Cancer resistance is often prevented through combination therapy, particularly when the combination drugs target different vulnerabilities in cancer cells [99]. Pro-senescence therapy appears particularly well-suited for this combination therapy because senescence is a stable phenotype that can be induced by various stresses [100]. Furthermore, the physiological functions of senescent cells differ significantly in metabolism, secretion, transcription, and epigenetics, and these differences can all become potential targets for vulnerability. Thus, combining pro-senescence drugs with drugs that selectively clear senescent cells (senolytic) will be a feasible and innovative combination therapy for cancer [12]. This combination approach has been confirmed in previous studies. For example, combining PTC028, an inhibitor of BMI1 that can suppress diffuse intrinsic pontine glioma (DIPG) by promoting tumor cell senescence, with senolytic ABT-263 can significantly enhance the tumor-killing effect of PTC028 and reduce its long-term adverse events in vivo [101]. In our study, knockdown of *FAM83D* induced different dominant sub-clusters in tumor cells, with the senescence-immunoactive signature preferentially enriched in the TC1 and cancer-associated pathways preferentially enriched in the TC3. Even though, the cellular senescence was tended to be activated in all sub-clusters, they might have different SASP secretion profiles to match their own characteristics. In our mouse model, we have not yet observed that the long-term presence of specific senescent sub-cluster would affect tumor recurrence. This may be attributed to the TC1, which appears to activate a strong immune response to enhance tumor clearance. Thus, regulating the activity of the immune system may be a way to overcome the “dark side” of senescence. To facilitate the clinical application of this treatment, further drug trials by designing inhibitors targeting FAM83D are needed to evaluate the long-term efficacy of this approach. Additionally, SASP represents a dynamic and evolving mixture of secreted factors, with its composition changing over time dependent on the initial trigger [102]. To date, longitudinal experiments to depict the dynamic evolution trajectory of SASP in glioma are still lacking. Notably, a previous study showed that 24-hour-collected condition medium from metformin- or simvastatin-treated cells could induce a senescence state in glioma [11], which aligned with the effect of 48-hour-collected condition medium following *FAM83D* knockdown. Therefore, characterizing therapy-specific SASP profiles at various time points should be a priority for advancing the next generation of pro-senescence therapies in glioma.

There are still some limitations of this study. First, we lack the observation of the interaction between FAM83D and the mitotic spindle in tumor cells during the dividing phase to better confirm our findings. To gain deeper insights, future studies should employ live-cell imaging or high-resolution immunofluorescence specifically depicting aberrant spindle morphology in FAM83D-deficient cells. Second, the evidence about ANXA1-FPR1/2 signaling as a mediator of macrophage polarization is not directly validated. Targeted perturbation of ANXA1 function will provide additional insights.

## Conclusions

In summary, this study underscores the critical role of FAM83D in regulating glioma cell senescence and shaping the tumor microenvironment with a specific impact on macrophage polarization (Fig. 8G). In general, knockdown of *FAM83D* in glioma cells disrupts normal cell division, leading to the accumulation of dsDNA in the cytoplasm. Accumulated dsDNA activates the cGAS-STING signaling pathway to induce glioma cell senescence. The SASP factors produced by senescent glioma cells can, on the one hand, induce senescence in neighboring non-senescent glioma cells; on the other hand, promote M1 polarization of macrophages to activate the immune response for suppressing glioma progression. Given the advantages of combination therapy, the pro-senescence strategy by targeting the FAM83D-cGAS/STING-SASP-TAMs axis holds great promise for the clinical management of glioma.

## Supplementary Information

Supplementary Material 1: Supplementary Figure 1. Atlas of human glioma, developmental human brain, and aging mouse brain. (A) UMAP plot of cells from GSE117891 and GSE182109 datasets colored by cell types. (B) UMAP plot of cells from the GSE120046 dataset colored by cell types (left) and proportion of each cell age across samples, respectively (right). (C) Projected cell types and ages in glioma by mapping the glioma dataset GSE182109 to the human brain developmental atlas. The diagram on the right shows the consistency between the original cell types and projected cell types in the microenvironment. (D) Distribution of projected cell ages in glioma dataset GSE182109. (E) Enrichment of replicative senescence associated signature, stress-induced senescence associated signature, stem cell associated signature, normal young adult upregulated signature, and normal old adult upregulated signature in the projected young versus projected old tumor cells from the GSE117891 and GSE182109 datasets. *p* value, two-sided unpaired Wilcoxon test. (F) GO biological process enrichment based on 94 common genes. q value, a corrected *p* value determined by the false discovery rate. (G) UMAP plot of cells from the GSE129788 dataset colored by cell types (top) and proportion of each cell age across samples respectively (bottom). (H) Functional classification of PSAG.

Supplementary Material 2: Supplementary Figure 2. Expression of *FAM83D* is closely related to the histopathological features of glioma and patient prognosis. (A) Differentially expressed levels of the top 15 core features selected by Mime in the normal young adult brain versus old adult brain. Young/old, the ratio of average expression level of gene in young adult brain to old adult brain from GTEx dataset. (B) Comparison of clinical features between the *FAM83D* high expression group and the low expression group in different glioma cohorts. *p* value, Chi-square test. (C) Comparison between clinical features and expression of *FAM83D* in different glioma cohorts. *p* value, two-sided unpaired Wilcoxon test for two groups, and Kruskal-Wallis test for multiple groups. (D) Relationship between expression of *FAM83D* and tumor grade or IDH mutation status in scRNA-seq. *p* value, two-sided unpaired t-test; error bars, mean ± SEM. (E) Multivariate Cox regression analysis of *FAM83D* based on the TCGA, CGGA.325, and CGGA.693 cohorts.

Supplementary Material 3: Supplementary Figure 3. Knockdown of *FAM83D* alters the transcriptional signature in tumor cells. (A) Relative expression of *FAM83D* in different cell lines under different conditions. Each data point represents one biological replicate (*n* = 4 replicates for U251 and LN229 cell lines, n = 6 replicates for GL261 cell line). *p* value, two-sided unpaired t-test; error bars, mean ± SEM. (B) Expression of *FAM83D* in RNA-seq. *p* value, two-sided unpaired Wilcoxon test. (C) Co-localization analysis of DAPI, FAM83D, and α-Tubulin based on Fig. 4D. The fluorescence intensity of each marker along a straight line (35 μm) passing through both the cytoplasm and the center of the nucleus is scaled to range 0-1. Closely spaced lines indicate co-localization between markers. (D) Representative WB image of p-p65, p65, p21, and p16 in U251 and LN229 under different conditions (top) and quantification of protein level normalized to the control group (bottom). Concentration of H-151 is 1 μM. Each data point represents one biological replicate (*n*=4 replicates for each cell line). *p* value, two-sided unpaired t-test; error bars, mean ± SEM. (E) Expression of senescence-related genes in RNA-seq of the U251 cell line. The red genes are representative SASP-related genes. (F) UMAP plot of cells from scRNA-seq colored by groups. (G) Violin plot showing the expression of classical markers in scRNA-seq. (H) CNV score for each cell in scRNA-seq. A higher score indicates more malignancy.

Supplementary Material 4: Supplementary Figure 4. Knockdown of *FAM83D* induces a more immunoactive microenvironment. (A) Enrichment scores of hallmark of cancer in different tumor cell sub-clusters. (B) Proportion of each tumor cell sub-cluster across samples, respectively. *p* value, Chi-square test. (C) Enrichment scores of cellular senescence and cGAS-STING signaling pathway in different tumor cell sub-clusters. *p* value, Kruskal-Wallis test. (D) UMAP plot of T cells from scRNA-seq colored by sub-clusters. (E) Violin plot showing the expression of classical T-cell markers in scRNA-seq. (F) Proportion of each T cell sub-cluster across samples, respectively. *p* value, Chi-square test. (G) Enrichment scores of cytotoxic and exhausted states in CD8 T cells between different groups. *p* value, two-sided unpaired Wilcoxon test. (H) Enrichment scores of regulatory state in CD4 T cells between different groups. *p* value, two-sided unpaired Wilcoxon test.

Supplementary Material 5: Supplementary Figure 5. Spatial transcriptome and bulk transcriptome cohort of glioma. (A) Annotation of spots in the spatial transcriptome cohort of glioma colored by MPs. (B) Annotation of spots in the spatial transcriptome cohort of glioma colored by layers. (C) Proportion of each MP across layers, respectively, in the spatial transcriptome of glioma. (D) Correlation between the expression of *FAM83D* and the proportion of non-inflammatory macrophage in the spatial transcriptome of glioma. *p* value, Pearson’s correlation test. (E) Expression of *FAM83D* in RNA-seq of Xiangya glioma cohort. *p* value, two-sided unpaired Wilcoxon test. (F) Comparison between clinical features and expression of *FAM83D* in the Xiangya glioma cohort. *p* value, two-sided unpaired Wilcoxon test for two groups, and Kruskal-Wallis test for multiple groups.

Supplementary Material 6: Table S1. Primers for qPCR.

## Data Availability

Bulk and Single-cell RNA sequencing data have been deposited in the National Genomics Data Centre (NGDC, https://ngdc.cncb.ac.cn/) under the accession number PRJCA055724 and PRJCA055734. This study did not generate original codes. All software and algorithms used in this study are publicly available. Any additional information required to reanalyze the data reported in the manuscript can be obtained from the corresponding author upon reasonable request.

## Abbreviations

ANXA1: Annexin A1
cGAS: Cyclic GMP-AMP synthase
CGGA: China Glioma Genome Atlas
DEGs: Differentially expressed genes
dsDNA: Double-stranded DNA
FAM83D: Family with sequence similarity 83 member D
GBM: Glioblastoma
GO: Gene ontology
GTEx: Genotype-Tissue Expression project
GW: Gestational week
KEGG: Kyoto encyclopedia of genes and genomes
Log2FC: Log2 fold change
MPs: Meta-programs
NMF: Non-negative matrix factorization
PSAG: Projected senescence associated signatures in glioma
SASP: Senescence-associated secretory phenotype
SA-β-gal: Senescence-associated β-galactosidase
scRNA-seq: Single-cell RNA sequencing
STING: Stimulator of interferon genes
TAMs: Tumor-associated macrophages
TC: Tumor sub-cluster
TCGA: The Cancer Genome Atlas Program
TME: Tumor microenvironment
