# Supplementary figures and images for: Targeting FAM83D triggers tumor cell senescence via cGAS-STING signaling activation and reprograms TAMs to combat glioma
Source: J Exp Clin Cancer Res. 2026 Feb 26;45:85. doi: 10.1186/s13046-026-03681-y (PMC13041050; doi:10.1186/s13046-026-03681-y)

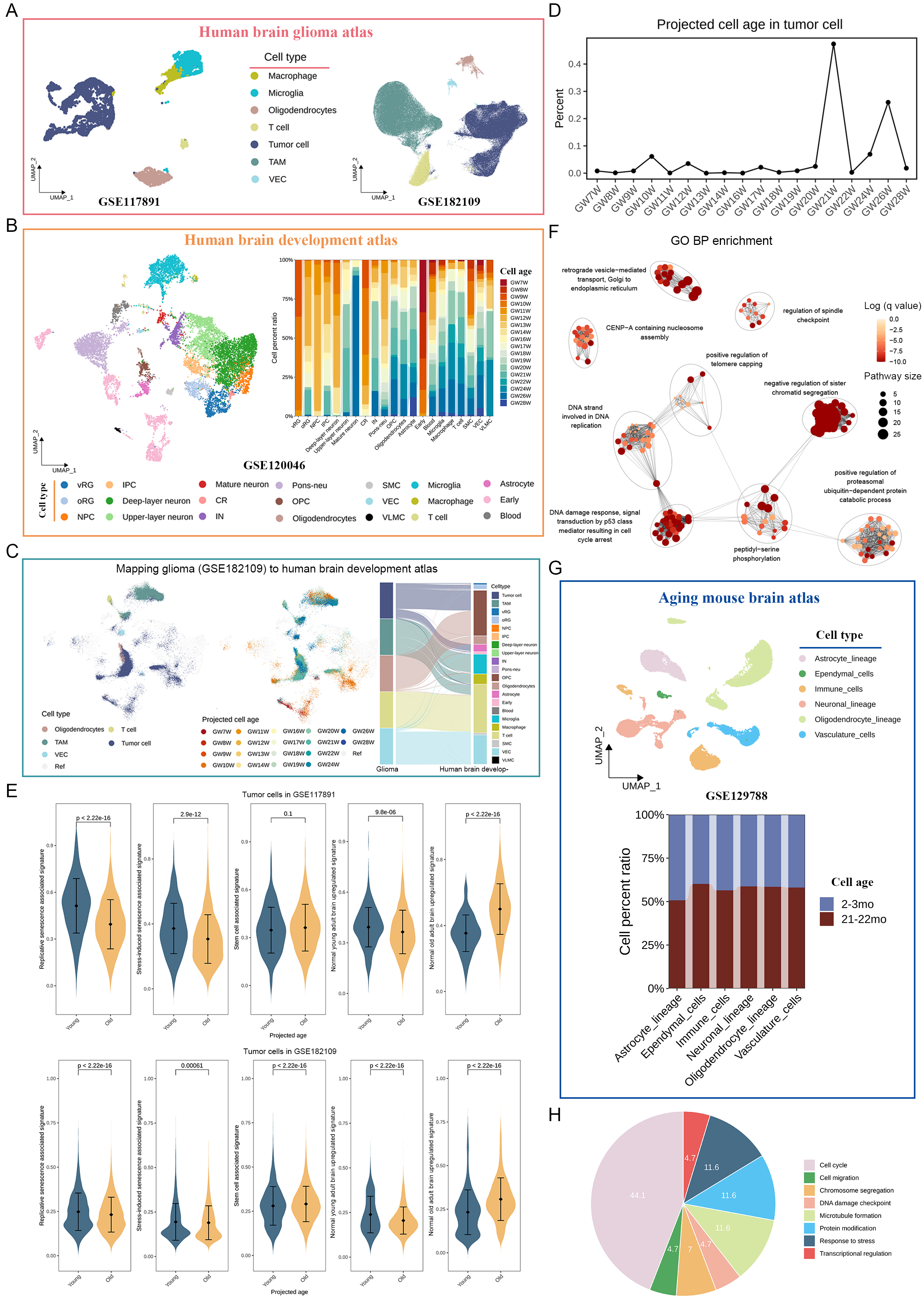

Supplement: Supplementary file 1 — Supplementary Material 1: Supplementary Figure 1. Atlas of human glioma, developmental human brain, and aging mouse brain. (A) UMAP plot of cells from GSE117891 and GSE182109 datasets colored by cell types. (B) UMAP plot of cells from the GSE120046 dataset colored by cell types (left) and proportion of each cell age across samples, respectively (right). (C) Projected cell types and ages in glioma by mapping the glioma dataset GSE182109 to the human brain developmental atlas. The diagram on the right shows the consistency between the original cell types and projected cell types in the microenvironment. (D) Distribution of projected cell ages in glioma dataset GSE182109. (E) Enrichment of replicative senescence associated signature, stress-induced senescence associated signature, stem cell associated signature, normal young adult upregulated signature, and normal old adult upregulated signature in the projected young versus projected old tumor cells from the GSE117891 and GSE182109 datasets. p value, two-sided unpaired Wilcoxon test. (F) GO biological process enrichment based on 94 common genes. q value, a corrected p value determined by the false discovery rate. (G) UMAP plot of cells from the GSE129788 dataset colored by cell types (top) and proportion of each cell age across samples respectively (bottom). (H) Functional classification of PSAG. [file 13046_2026_3681_MOESM1_ESM.jpg]

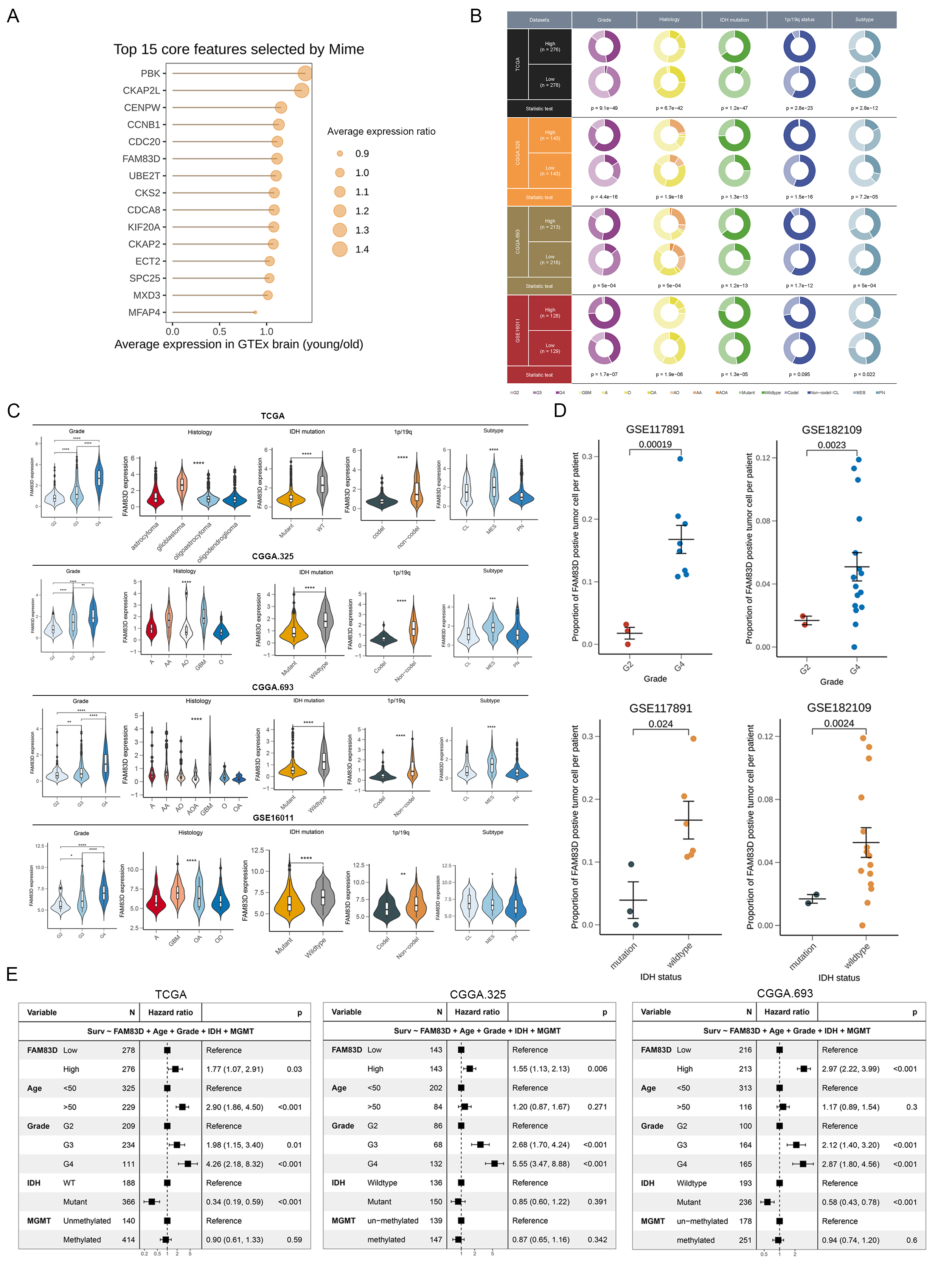

Supplement: Supplementary file 2 — Supplementary Material 2: Supplementary Figure 2. Expression of FAM83D is closely related to the histopathological features of glioma and patient prognosis. (A) Differentially expressed levels of the top 15 core features selected by Mime in the normal young adult brain versus old adult brain. Young/old, the ratio of average expression level of gene in young adult brain to old adult brain from GTEx dataset. (B) Comparison of clinical features between the FAM83D high expression group and the low expression group in different glioma cohorts. p value, Chi-square test. (C) Comparison between clinical features and expression of FAM83D in different glioma cohorts. p value, two-sided unpaired Wilcoxon test for two groups, and Kruskal-Wallis test for multiple groups. (D) Relationship between expression of FAM83D and tumor grade or IDH mutation status in scRNA-seq. p value, two-sided unpaired t-test; error bars, mean ± SEM. (E) Multivariate Cox regression analysis of FAM83D based on the TCGA, CGGA.325, and CGGA.693 cohorts. [file 13046_2026_3681_MOESM2_ESM.jpg]

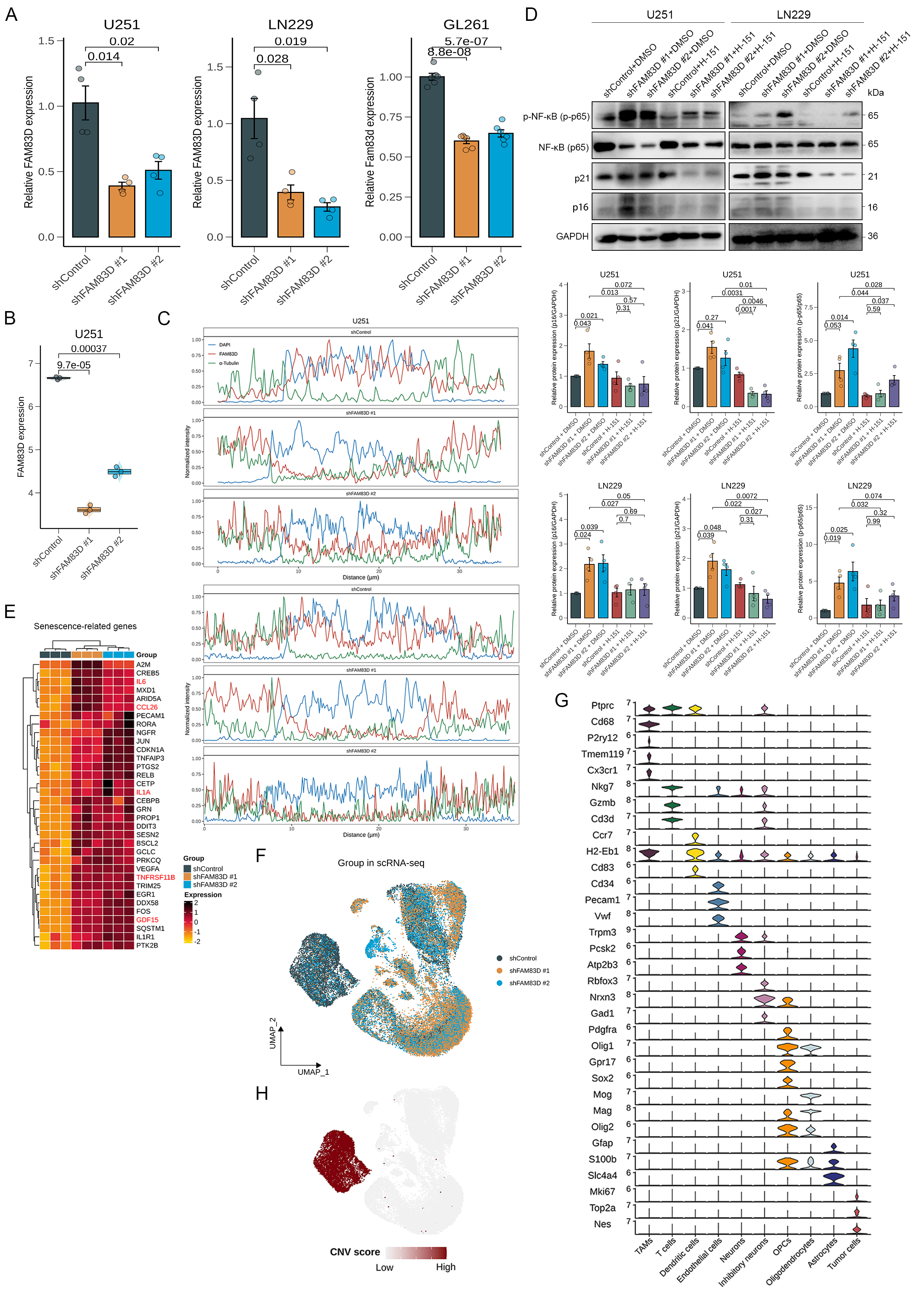

Supplement: Supplementary file 3 — Supplementary Material 3: Supplementary Figure 3. Knockdown of FAM83D alters the transcriptional signature in tumor cells. (A) Relative expression of FAM83D in different cell lines under different conditions. Each data point represents one biological replicate (n = 4 replicates for U251 and LN229 cell lines, n = 6 replicates for GL261 cell line). p value, two-sided unpaired t-test; error bars, mean ± SEM. (B) Expression of FAM83D in RNA-seq. p value, two-sided unpaired Wilcoxon test. (C) Co-localization analysis of DAPI, FAM83D, and α-Tubulin based on Fig. 4D. The fluorescence intensity of each marker along a straight line (35 μm) passing through both the cytoplasm and the center of the nucleus is scaled to range 0-1. Closely spaced lines indicate co-localization between markers. (D) Representative WB image of p-p65, p65, p21, and p16 in U251 and LN229 under different conditions (top) and quantification of protein level normalized to the control group (bottom). Concentration of H-151 is 1 μM. Each data point represents one biological replicate (n=4 replicates for each cell line). p value, two-sided unpaired t-test; error bars, mean ± SEM. (E) Expression of senescence-related genes in RNA-seq of the U251 cell line. The red genes are representative SASP-related genes. (F) UMAP plot of cells from scRNA-seq colored by groups. (G) Violin plot showing the expression of classical markers in scRNA-seq. (H) CNV score for each cell in scRNA-seq. A higher score indicates more malignancy. [file 13046_2026_3681_MOESM3_ESM.jpg]

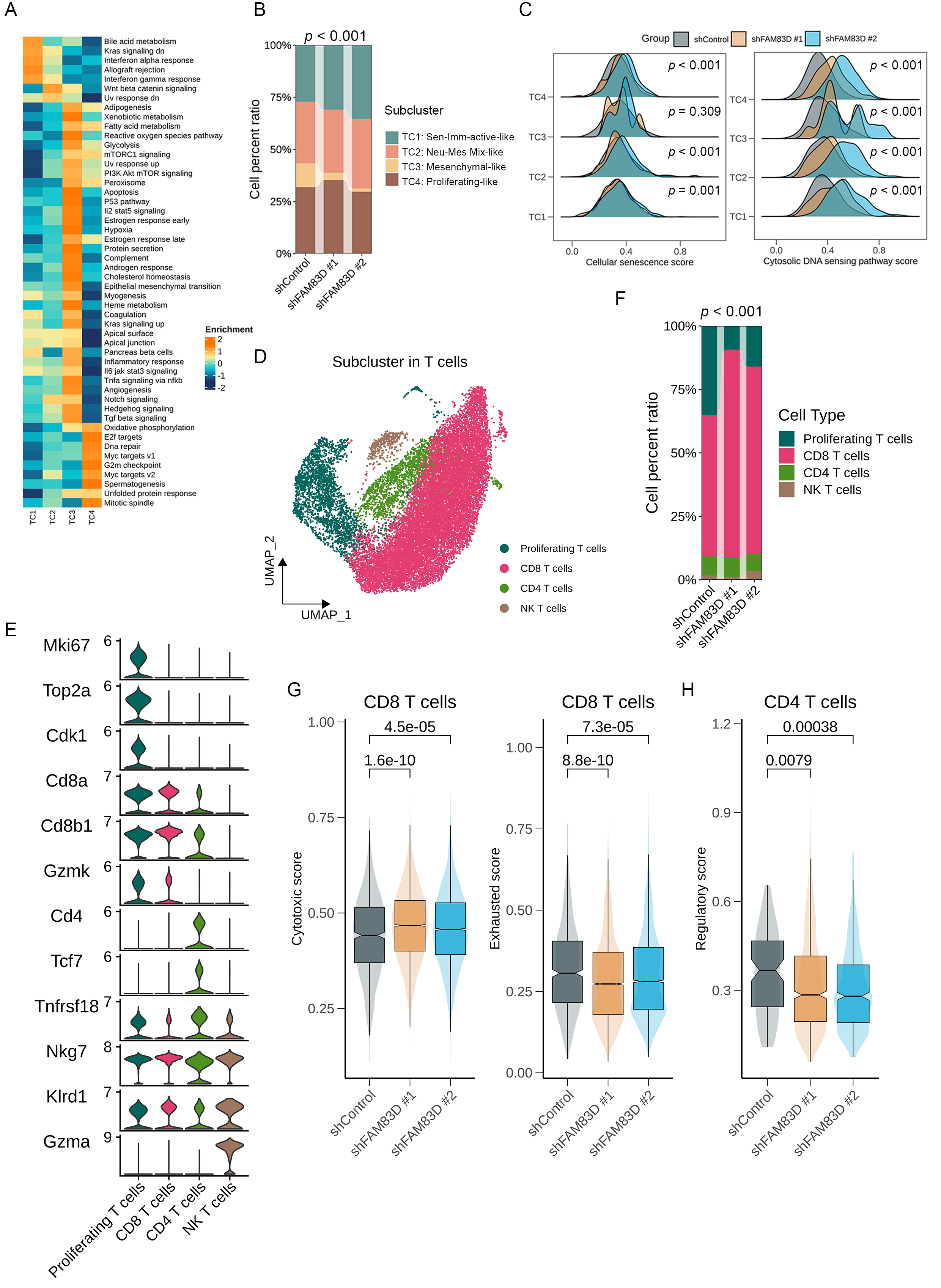

Supplement: Supplementary file 4 — Supplementary Material 4: Supplementary Figure 4. Knockdown of FAM83D induces a more immunoactive microenvironment. (A) Enrichment scores of hallmark of cancer in different tumor cell sub-clusters. (B) Proportion of each tumor cell sub-cluster across samples, respectively. p value, Chi-square test. (C) Enrichment scores of cellular senescence and cGAS-STING signaling pathway in different tumor cell sub-clusters. p value, Kruskal-Wallis test. (D) UMAP plot of T cells from scRNA-seq colored by sub-clusters. (E) Violin plot showing the expression of classical T-cell markers in scRNA-seq. (F) Proportion of each T cell sub-cluster across samples, respectively. p value, Chi-square test. (G) Enrichment scores of cytotoxic and exhausted states in CD8 T cells between different groups. p value, two-sided unpaired Wilcoxon test. (H) Enrichment scores of regulatory state in CD4 T cells between different groups. p value, two-sided unpaired Wilcoxon test. [file 13046_2026_3681_MOESM4_ESM.jpg]

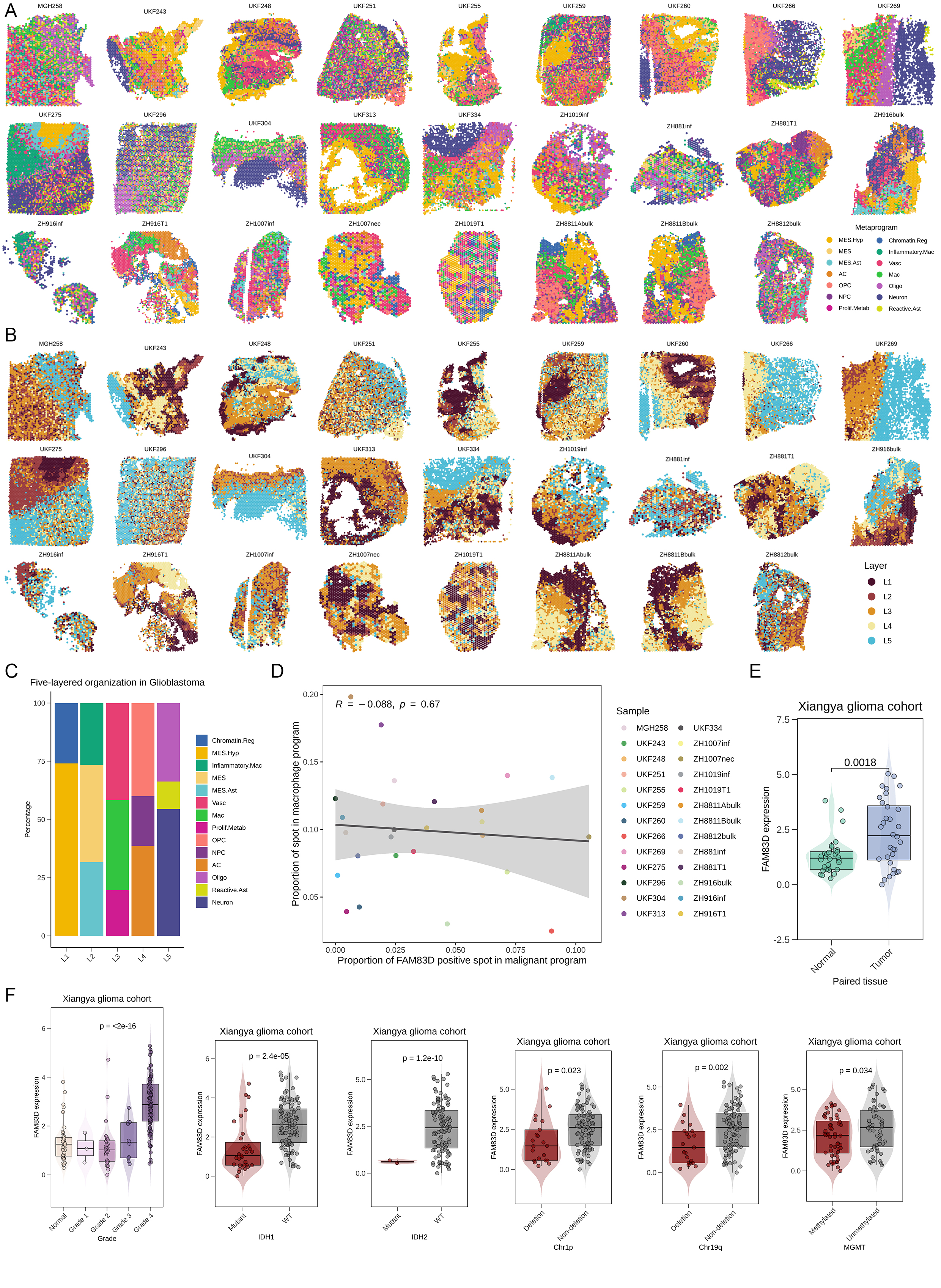

Supplement: Supplementary file 5 — Supplementary Material 5: Supplementary Figure 5. Spatial transcriptome and bulk transcriptome cohort of glioma. (A) Annotation of spots in the spatial transcriptome cohort of glioma colored by MPs. (B) Annotation of spots in the spatial transcriptome cohort of glioma colored by layers. (C) Proportion of each MP across layers, respectively, in the spatial transcriptome of glioma. (D) Correlation between the expression of FAM83D and the proportion of non-inflammatory macrophage in the spatial transcriptome of glioma. p value, Pearson’s correlation test. (E) Expression of FAM83D in RNA-seq of Xiangya glioma cohort. p value, two-sided unpaired Wilcoxon test. (F) Comparison between clinical features and expression of FAM83D in the Xiangya glioma cohort. p value, two-sided unpaired Wilcoxon test for two groups, and Kruskal-Wallis test for multiple groups. [file 13046_2026_3681_MOESM5_ESM.jpg]
